# Single-nucleus multi-omics of Parkinson’s disease reveals a glutamatergic neuronal subtype susceptible to gene dysregulation via alteration of transcriptional networks

**DOI:** 10.1186/s40478-024-01803-1

**Published:** 2024-07-02

**Authors:** E. Keats Shwab, Daniel C. Gingerich, Zhaohui Man, Julia Gamache, Melanie E. Garrett, Gregory E. Crawford, Allison E. Ashley-Koch, Geidy E. Serrano, Thomas G. Beach, Michael W. Lutz, Ornit Chiba-Falek

**Affiliations:** 1 Division of Translational Brain Sciences, Department of Neurology, Duke University School of Medicine, Duke University Medical Center, Durham, NC 27710 USA; 2 Center for Genomic and Computational Biology, Duke University Medical Center, Durham, NC 27708 USA; 3 Duke Molecular Physiology Institute, Duke University Medical Center, Durham, NC 27701 USA; 4 Division of Medical Genetics, Department of Pediatrics, Duke University Medical Center, Durham, NC 27708 USA; 5 Center for Advanced Genomic Technologies, Duke University Medical Center, Durham, NC 27708 USA; 6 Department of Medicine, Duke University Medical Center, Durham, NC 27708 USA; 7 Banner Sun Health Research Institute, Sun City, AZ 85351 USA

**Keywords:** Parkinson’s disease, Single-nucleus (sn)RNA-seq, snATAC-seq, Candidate *cis* regulatory element (cCRE), Regulatory networks, Glutamatergic neurons, Transcription factor binding sites, Functional SNVs and indels

## Abstract

The genetic architecture of Parkinson’s disease (PD) is complex and multiple brain cell subtypes are involved in the neuropathological progression of the disease. Here we aimed to advance our understanding of PD genetic complexity at a cell subtype precision level. Using *parallel* single-nucleus (sn)RNA-seq and snATAC-seq analyses we simultaneously profiled the transcriptomic and chromatin accessibility landscapes in temporal cortex tissues from 12 PD compared to 12 control subjects at a granular single cell resolution. An integrative bioinformatic pipeline was developed and applied for the analyses of these snMulti-omics datasets. The results identified a subpopulation of cortical glutamatergic excitatory neurons with remarkably altered gene expression in PD, including differentially-expressed genes within PD risk loci identified in genome-wide association studies (GWAS). This was the only neuronal subtype showing significant and robust overexpression of *SNCA*. Further characterization of this neuronal-subpopulation showed upregulation of specific pathways related to axon guidance, neurite outgrowth and post-synaptic structure, and downregulated pathways involved in presynaptic organization and calcium response. Additionally, we characterized the roles of three molecular mechanisms in governing PD-associated cell subtype-specific dysregulation of gene expression: (1) changes in cis-regulatory element accessibility to transcriptional machinery; (2) changes in the abundance of master transcriptional regulators, including YY1, SP3, and KLF16; (3) candidate regulatory variants in high linkage disequilibrium with PD-GWAS genomic variants impacting transcription factor binding affinities. To our knowledge, this study is the first and the most comprehensive interrogation of the multi-omics landscape of PD at a cell-subtype resolution. Our findings provide new insights into a precise glutamatergic neuronal cell subtype, causal genes, and non-coding regulatory variants underlying the neuropathological progression of PD, paving the way for the development of cell- and gene-targeted therapeutics to halt disease progression as well as genetic biomarkers for early preclinical diagnosis.

**Supplementary Information:**

The online version contains supplementary material available at 10.1186/s40478-024-01803-1.

## Introduction

Parkinson’s disease (PD) is the second most common and the fastest-growing neurodegenerative disorder [1, 2], with a complex etiology involving environmental and genetic factors. Mutations in several genes such as, alpha-synuclein (*SNCA*), *LRRK2*, *GBA*, *DJ-1* (*PARK7*), *PINK1*, and *PRKN*, were discovered in families with the rare familial form of PD (fPD) [3, 4]. However, the genetic complexity of the more common non-mendelian PD is yet to be fully understood. Large-scale genome-wide association studies (GWAS) have identified more than 100 single nucleotide variants (SNV) [5, 6] and structural variants (SV) [7, 8] associated with increased risk for PD, pointing at disease-associated chromosomal regions but not directly identifying causal variants and/or genes driving PD pathogenesis. Moreover, several PD-associated genetic variants, including short tandem repeat variants (STRs), have been suggested to have functional effects on gene expression [9–12]. However, the mechanisms through which they affect gene expression in the context of PD have not yet been described. Recent technological advancements have facilitated more granulated analyses on the molecular phenotypes of PD at the single-cell level. Single-nucleus RNA sequencing (snRNA-seq) studies have examined cellular heterogeneity of the substantia nigra (SN) and cortical regions [13, 14], and identified a sub-population of SN dopaminergic neurons that is selectively vulnerable to degeneration in PD [15]. Other studies suggested that microglial-mediated neuroinflammation [16] and astrocyte-mediated modulation of neuronal health [17] play roles in PD pathogenesis. More recently, an snRNA-seq analysis identified PD-associated gene expression changes of broad cell types in the SN, and following data integration with snATAC-seq and Chromatin Immunopreciptation (ChIP)-seq discovered candidate *cis* regulatory elements (cCREs) linked to PD-associated differentially expressed genes (DEGs) [18]. However, these emerging studies represent only the first steps towards a comprehensive exploration of the genetic architecture of PD. While in-depth transcriptomic analyses of PD have only been carried out on the broad cell type level, evidence suggests that specific cellular subtypes play important differential roles in the disease [15]. Thus, to gain meaningful understanding of the molecular mechanisms driving PD pathogenesis it is imperative to examine the genetics of PD at a more precise resolution within specialized cell subtypes. An additional gap in knowledge arises from the fact that the few PD single-cell transcriptomic analyses [13–15, 18] have focused only on the SN where early neurodegeneration is most severe and thus may obfuscate distinction between disease-driving events vs. consequences of neurodegenerative processes. As cortical brain regions have been shown to play an important role in PD pathology in later stages as the disease progresses [19–21], an examination of gene expression in cortical tissue with no to mild pathological perturbation provides a valuable window to capture molecular changes occurring prior to neurodegenerative processes, as well as offering new insights into genetic factors contributing to disease progression.

**Fig. 1:**
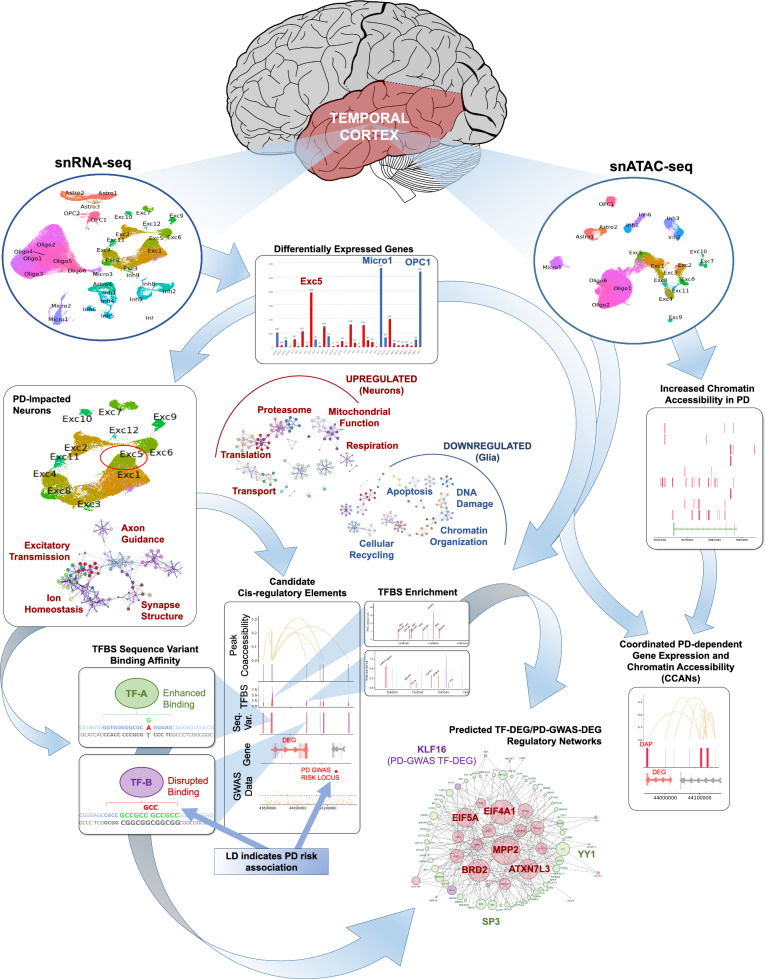
Patterns and predicted mechanisms of gene regulation in cell subtypes of the temporal cortex in PD. Summary of analysis pipeline and major findings. snRNA-seq analysis of temporal cortex nuclei from normal and PD donors revealed PD-dependent differential gene expression in cell subtype clusters, with strong polarization towards up- or downregulation in specific clusters. Exc5, Micro1 and OPC1 clusters stood out as having greater numbers of differentially-expressed genes (DEGs) compared to other clusters. Upregulated DEGs were enriched for functional pathways relating to translation, proteasomal protein degradation, mitochondrial function, cellular respiration, and intracellular transport. Downregulated DEGs were enriched for functional pathways relating to chromatin organization, DNA damage response, regulation of apoptosis, and cellular recycling. Exc5 was characterized by positive marker genes enriched for functions relating to excitatory neurotransmission, postsynaptic structure, regulation of intracellular ion homeostasis, and neuron development including axon guidance. snATAC-seq analysis revealed overall increased chromatin accessibility in PD for each cell subtype cluster examined. In some cases, differentially-accessible peaks (DAPs) formed networks (CCANs) with peaks that were coaccessible to regulatory regions of DEGs (candidate cis-regulatory elements; cCREs). Enrichment of transcription factor binding sites (TFBSs) was identified within cCREs of numerous GWAS DEGs. cCREs of certain PD GWAS-DEGs were found to be enriched for binding of high numbers of DEG TFs, including *MPP2*, *BRD2*, *ATXN7L3*, *EIF4A1*, and *EIF5A*, among others. DEG-encoded TFs (TF-DEGs) found to be enriched for binding within cCREs of multiple GWAS DEGs included YY1, SP3, and KLF16, which was itself also encoded by a GWAS DEG, among others. Additionally, sequence variants with high LD to GWAS-defined PD risk alleles were identified within cCREs of GWAS DEGs predicted to enhance or disrupt TF binding, including binding of several TF-DEGs (including YY1, SP3 and KLF16) predicted to regulate multiple PD GWAS-DEGs

Here we conducted the largest single-cell-sequencing-based analysis of PD to date, and the first in the human temporal cortex (TC), examining integrated transcriptomic and chromatin accessibility data at a cell-subtype level. Moreover, this is the first study evaluating the functional impact of SNVs, indels, and STRs on the binding affinity of transcription factors co-expressed in the same particular cell subtypes as the candidate PD-DEGs. Collectively, our goal was to discover cell subtype-specific gene expression changes associated with PD and their linked regulatory elements, variants, and transcriptional networks. We further aimed to identify and characterize cellular subtype/s exhibiting selective susceptibility to gene dysregulation in PD progression. Figure 1 describes our overall approach towards accomplishing these aims. Briefly, we defined in *parallel* gene expression (snRNA-seq) and chromatin accessibility (snATAC-seq) profiles of more than 200,000 nuclei extracted from 12 PD and 12 normal TC tissue samples. This enabled us to identify and characterize a sub-population of glutamatergic excitatory neurons that showed remarkable PD-associated dysregulation of transcriptional programs compared to all other neuronal subtypes. Finally, we integrated our data with PD-GWAS risk loci and transcription factor binding motif datasets to obtain new insights into the potential mechanisms underlying cell subtype-specific dysregulation of gene expression in PD.

## Results

### Characterization of cell types and subtypes in the human temporal cortex (TC) of individuals with PD and neurologically normal controls in snRNA-seq dataset

Nuclei were isolated from frozen post-mortem human TC tissues of 12 PD and 12 normal control individuals, 6 females and 6 males from each diagnosis group (Table 1 and Table S1 summarize the demographic and neuropathological phenotypes).

**Table 1:** Demographics and pathology summary of study cohort

Sample ID	Diagnosis	Sex	Age	PMI	USSLB Stage	McKeith Score		
						SN	Amygdala	TC
963	Normal	F	82	8.12	NA	NA	NA	NA
984	Normal	F	65	1.96	NA	NA	NA	NA
1545	Normal	F	64	10.67	NA	NA	NA	NA
1600	Normal	F	85	12.97	NA	NA	NA	NA
1670	Normal	F	87	18	NA	NA	NA	NA
1690	Normal	F	84	4.42	NA	NA	NA	NA
99	Normal	M	85	2	NA	NA	NA	NA
196	Normal	M	75	18.88	NA	NA	NA	NA
542	Normal	M	82	3.25	NA	NA	NA	NA
676	Normal	M	60	9.5	NA	NA	NA	NA
688	Normal	M	63	7	NA	NA	NA	NA
1557	Normal	M	90	4	NA	NA	NA	NA
01–38	PD	F	80	7.5	III	3	4	0
05–17	PD	F	84	2	IIa	NA	2	0
96–49	PD	F	87	2	NA	NA	NA	NA
99–09	PD	F	77	2.66	IIa	3	2	0
99–17	PD	F	83	12	III	2	4	1
99–38	PD	F	78	1.75	III	2	4	1
11–90	PD	M	78	2.75	III	3	4	1
12–29	PD	M	86	3	IV	4	4	2
13–11	PD	M	81	3.62	III	3	3	1
96–36	PD	M	88	2	NA	NA	NA	NA
98–29	PD	M	77	4	IV	2	3	3
99–63	PD	M	85	2.66	III	3	3	1

PD, Parkinsons’s disease; F, female; M, male; PMI, post-mortem interval; USSLB, unified staging system for Lewy body disorders; SN, substantia nigra; TC, temporal cortex; NA, not available

**Fig. 2:**
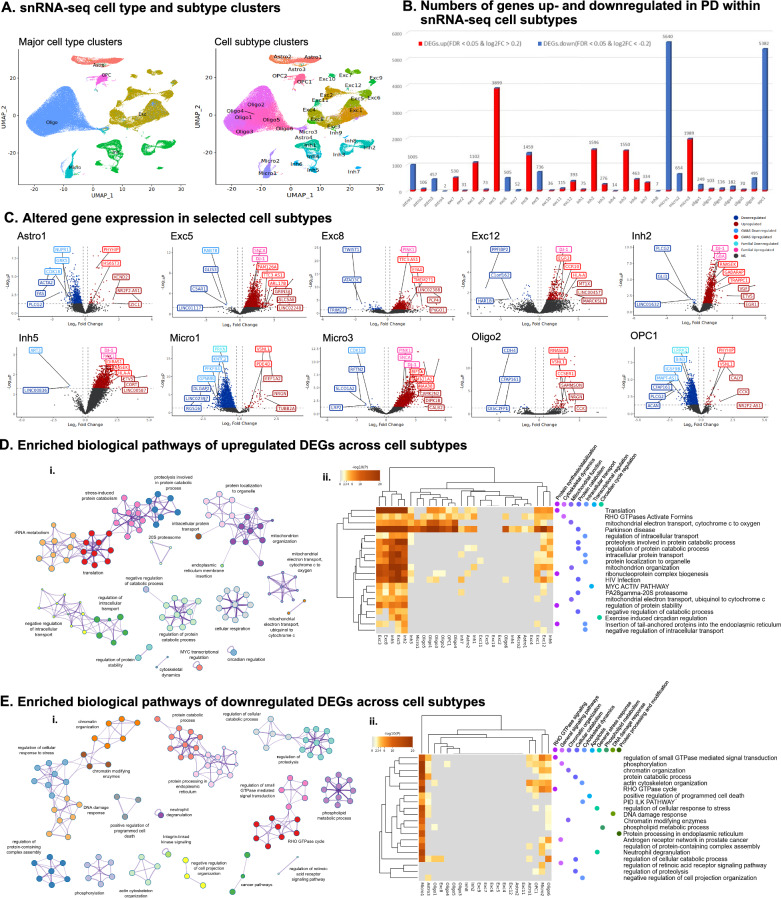

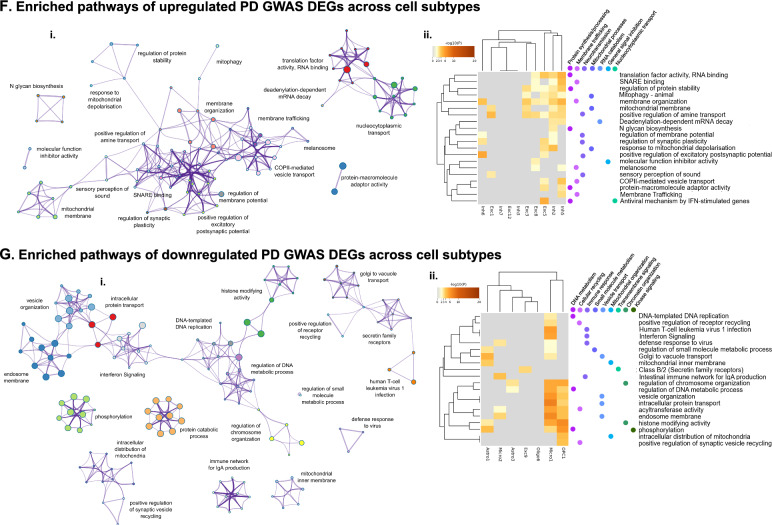
Differential gene expression in major cell types and subtype clusters. **A** UMAP dimensional reduction plot of major cell types: Astrocytes (Astro), Excitatory neurons (Exc), Inhibitory neurons (Inh), Microglia (Micro), Oligodendrocytes (Oligo), and Oligodendrocyte Precursor Cells (OPC) (left) and 36 cell subtype clusters (right) based on snRNA-seq data. **B** Histogram showing the number of DEGs with |log_2_FC| > 0.2 for each cell subtype cluster. Proportions of upregulated DEGs are indicated in red, while the proportions of downregulated DEGs are indicated in blue for each cluster. Total number of DEGs is indicated above each bar. **C** Unbiased volcano plots for selected cell subtype clusters, representing astrocyte (Astro), excitatory neuron (Exc), inhibitory neuron (Inh), microglia (Micro), oligodendrocyte (Oligo), and oligodendrocyte precursor (OPC) cell subtypes based on snRNA-seq clustering. Log_2_ fold change (FC) between PD and normal control samples is plotted against –log_10_ *p* value (FDR). Points representing DEGs with statistically significant (FDR < 0.05) upregulation in PD are shown in dark red while DEGs with significant downregulation are shown in dark blue. Genes without significantly differential expression are shown as gray points. The three DEGs with the highest absolute fold change (|log_2_FC| > 0.2) in the up- and downregulated categories are labeled in dark red and dark blue, respectively. The three familial PD genes with the highest absolute log_2_FC in the up- and downregulated categories are labeled in pink and teal, respectively. The three DEGs within 500 kb of PD-associated SNPs previously identified in GWAS (PD GWAS-DEGs) with the highest absolute log_2_FC in the up- and downregulated categories are labeled in bright red and bright blue, respectively. **D** Metascape analysis of enriched pathways among all upregulated DEGs across cell subtype clusters. Network diagrams (i) indicate shared genes among enriched biological pathways. Node colors indicate clusters with similar functionality (indicated by cluster labels) and node sizes are proportional to the number of marker genes included in the pathway. Widths of lines linking pathway nodes are proportional to the number of shared genes between the two linked pathways. Heatmaps (ii) indicate the top 20 enriched biological pathway terms across cell subtype clusters. Darker-colored rectangles indicate greater statistical significance (proportional to Log_10_ *p* value) of pathway enrichment. Pathways are labeled with dots indicating membership in broad-functional based clusters based on clustering in associated networks. Cladogram connecting lines indicate similarity of pathways (left) and clusters (top) with regard to shared DEGs in associated pathways. **E** Metascape analysis of enriched pathways among all downregulated DEGs across cell subtype clusters. Format is as in panel D. **F** Metascape analysis of enriched pathways among only upregulated PD GWAS-DEGs across cell subtype clusters. Format is as in panel D. **G** Metascape analysis of enriched pathways among only downregulated PD GWAS-DEGs across cell subtype clusters. Format is as in panel D

After quality control (QC) filtering, a total of 208,081 nuclei from all 24 TC samples were retained for snRNA-seq (Table S1). Cell types were annotated by the label transfer method [22] following gene expression library sequencing, using a pre-annotated reference snRNA-seq dataset [23]. Annotations were validated by examination of cell type-specific marker gene expression [24] (Fig. S1). Six major cell types were identified including: astrocytes (Astro), excitatory neurons (Exc), inhibitory neurons (Inh), microglia (Micro), oligodendrocytes (Oligo), and oligodendrocyte precursor cells (OPC; Fig. 2A). Oligodendrocytes were the most prevalent cell type in our dataset (96,812 nuclei in total), followed by excitatory neurons (50,590 nuclei), whereas OPCs were the least prevalent major cell type (9564 nuclei). Vascular and leptomeningeal cells represented less than 1% of recovered nuclei in our dataset and were therefore excluded from downstream analyses. Recovery of sufficient quantities of this cell type would likely require sample pooling or additional enrichment procedures to enable cell dissociation from the vessel basement membrane [25, 26]. Following dimensional reduction by principal component analysis (PCA) and uniform manifold approximation and projection (UMAP), Louvain community detection was used to delineate 36 nuclei clusters with distinct gene expression profiles (Fig. 2A, Table S2) and representing subtypes of the six major cell types. Cell subtypes were each given a unique label according to their most common cell type (e.g., the 12 clusters of excitatory neurons were labeled Exc1-Exc12; Fig. 2A, Table S2). To check for donor-based batch effects, we examined the distribution of nuclei across subtype clusters for each donor sample. Donor samples of both Normal and PD groups overall showed qualitatively even distribution across cell subtype clusters (Fig. S2).

PD pathology is characterized by neuronal cell death [27]. Thus, we compared the proportions of major cell types and subtypes (number of nuclei from a particular donor sample belonging to a particular cell type/subtype divided by total number of nuclei for the same sample) between PD and control samples. The comparison analysis was performed using the Mixed-Effects Association of Single Cells (MASC) algorithm [28] with age, sex, *postmortem*-interval (PMI), number of nuclei after QC filtering, median genes per cell, and average library size as fixed effects and sample donor ID included as a random effect. We did not identify any significant changes in either major cell type or cell subtype cluster proportions between the PD and the control groups (Fig. S3A), as expected according the low McKeith scores in the TC tissues of the PD group (Table 1 and S1).

### Cell-type and subtype-specific differential gene expression in PD

To examine differential gene expression between PD and the normal control samples using the snRNA-seq data, we employed the NEgative Binomial mixed model Using a Large-sample Approximation (NEBULA) algorithm [29]. First, we analyzed the contributions of 41 metadata variables (listed in Table S1) to sample variance and found that the number of nuclei after QC filtering, median number of genes per cell, and average library size contribute significantly to the variability in gene expression. Thus, we included these variables as fixed effects in our NEBULA model, along with donor age, sex, and sample collection PMI. In addition, sample donor ID was included as a random effect to account for donor-specific variance. Changes in gene expression were compared between PD and control for each of the 6 major cell types and each of the 36 subtype clusters, using a gene-level false discovery rate (FDR)-corrected *p* value significance threshold of < 0.05 and a log_2_ fold-change (FC) of ≥ 0.2 to define differentially expressed genes (DEGs). Among the major cell types, microglia produced the highest number of DEGs (n = 4849), while excitatory neurons produced the lowest number with only 3 DEGs (Fig. S4A). Of the major cell types, excitatory neurons were divided into the highest number of subtype clusters (n = 12) which also tended to be clearly delineated in UMAP space (Fig. 2A), indicating a high degree of diversity among cell subtypes that may diminish the utility of aggregate analyses. Variability between clusters in this case may have overwhelmed variability between disease states. In contrast, microglia separated into only 3 clusters, with the bulk of nuclei belonging to the Micro1 cluster (Fig. 2A, Table S2). The fPD gene *DJ-1 (PARK7)* was identified as a DEG in both inhibitory neurons and oligodendrocytes, and *PRKN* and *LRRK2* were DEGs in microglia and OPCs, respectively (Fig. S4A).

A granulated analysis at the cell subtype level revealed a total of 11,612 unique DEGs, with the highest numbers of DEGs in Exc5, Micro1, and OPC1 clusters (n = 3899, 5640, and 5382, respectively; Fig. 2B, Table S3). On the other hand, there were no DEGs in the Inh9 and OPC2 clusters. Noteworthily, while Exc5 and other Exc clusters (e.g., Exc3 and Exc8) showed numerous DEGs, very few DEGs were found in Exc neurons when the analysis was performed at a broad cell type level, suggesting roles for specific Exc subtypes in PD pathogenesis. Notably, we observed that in most cell subtypes the directionality of the DEGs was highly polarized, with the vast majority of DEGs being either upregulated or downregulated in PD in a given subtype (Fig. 2B, C, Fig. S4B). While directionality was generally consistent within clusters of a particular major cell type, in some cases specific subtype clusters showed a different directionality than the majority of clusters of that cell type. For example, the Exc6, 7, and 9 clusters showed primarily PD-associated decreases in gene expression while the other Exc clusters showed primarily increased expression (Fig. 2B, C, Fig. S4C). We then integrated the cell subtype DEGs dataset with 90 PD-GWAS loci [5] (Table S3). Out of a total of 2428 genes mapped to PD-GWAS regions, defined as ± 500 Kb of GWAS-SNVs, 628 were DEGs in at least one cell subtype (hereafter designated as PD GWAS-DEGs), including *SNCA* (Exc5 and Micro3), *GBA* (Inh2), and *LRRK2* (OPC1) (Fig. 2C, Fig. S4B, Table S3). *SNCA, GBA,* and *LRRK2* are not only PD GWAS loci in non-mendelian PD cases, but are also established fPD genes [4]. In addition to these three genes*,* in several clusters we identified other fPD DEGs, including, *DJ-1* (Exc5, Exc12, Inh1, Inh5, and Micro3), and *PINK1* (Exc8, Inh5, and Micro3) (Fig. 2C, Fig. S4B).

### Biological pathways enriched for cell-type specific PD-associated DEGs

Next, we assessed the biological significance of the identified DEGs using Metascape [30]. Enriched pathways for upregulated DEGs in the different cell subtypes were grouped into major functional categories relating to protein synthesis and stabilization, protein catabolism, intracellular transport, mitochondrial function and cellular respiration, transcriptional regulation, circadian signaling, and cytoskeletal dynamics (Fig. 2Di). Upregulation of these pathways was most strongly observed among neuronal subtypes, but was also detected in oligodendrocyte and microglia clusters (Fig. 2Dii). Downregulated DEGs across cell subtypes were enriched in pathways relating to DNA damage response, cellular stress response (including regulation of apoptosis), chromatin organization, catabolic processes (protein and other types), RHO GTPase signaling, cytoskeletal dynamics, kinase signaling, protein complex assembly, cancer pathways, and retinoic acid signaling (Fig. 2Ei). These downregulated pathways were enriched primarily in glial subtypes (Micro1, Micro2, Oligo6, Astro 1, Astro3, and OPC1; Fig. 2Eii).

To pinpoint pathways driving PD risk, we next examined pathway enrichment among PD GWAS-DEGs only. Upregulated pathways were enriched in categories relating to neurotransmission, membrane trafficking and organization, mitochondrial function and mitochondrial stress response, translation, and nucleocytoplasmic transport (Fig. 2Fi). Shared enriched pathways for upregulated PD GWAS-DEGs were found exclusively among neuronal subtypes (Fig. 2Fii), suggesting neurons as the primary PD drivers. Furthermore, these pathways were also associated with other PD-related functions, including protein synthesis [31–34] and catabolism [35–38], and mitochondrial function [39–43]. Downregulated PD GWAS-DEGs were enriched in pathways of diverse categories including DNA metabolism, immune response, cellular recycling pathways, mitochondrial organization, and vesicle transport (Fig. 2Gi). The analysis pointed to a decrease in stress-responsive functions, including pathways associated with the DNA damage response, in accordance with recent work highlighting the importance of DNA repair and other stress-response mechanisms in PD [44]. Downregulated pathways were enriched in glial cells, most prominently within the Micro1 and OPC1 clusters, including immune response pathways specific to microglia (Fig. 2Gii). These results suggest suppression of pathways regulating DNA metabolism and chromatin structure, cellular recycling, and immune-based stress responses within glial subtypes as drivers of PD. We also described the most highly enriched up- and downregulated PD GWAS-DEG pathways for individual cell subtype clusters (Fig. S5A and B, respectively).

### Disproportionate impact of a specific glutamatergic excitatory neuron subtype on PD pathological progression

**Fig. 3:**
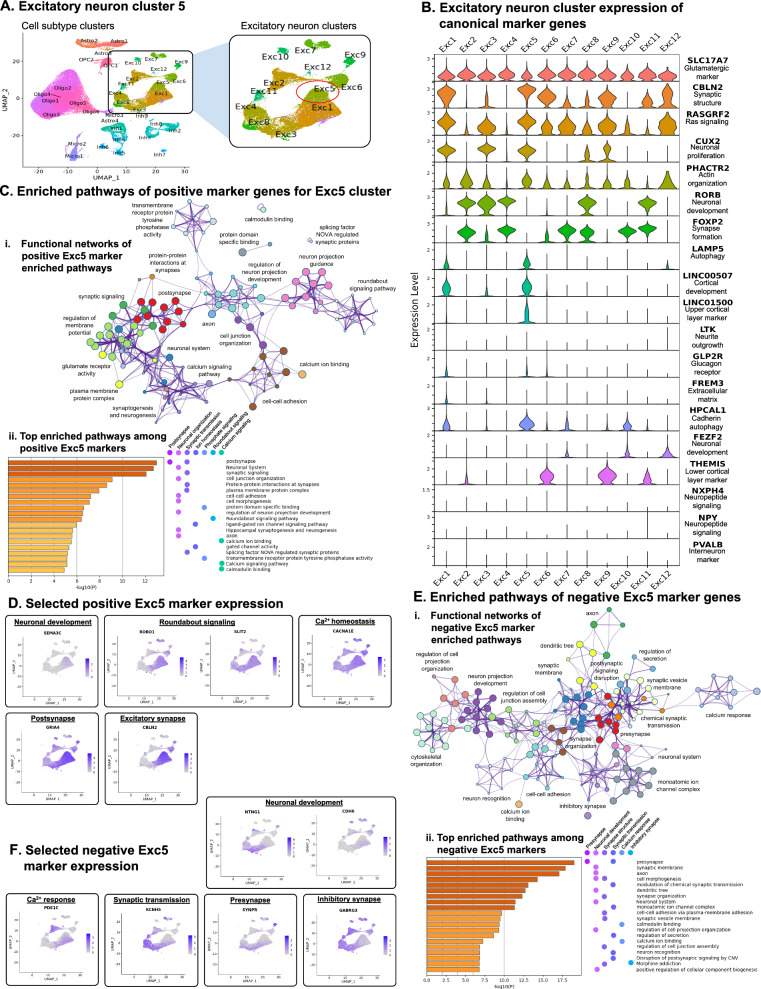
Characterization of excitatory neuron subtype cluster Exc5 by differential gene expression. **A** UMAP dimensional reduction plot of 36 cell subtypes in snRNA-seq dataset. Boxed area and associated enlarged panel indicates excitatory neuron (Exc) subtypes and the Exc5 cluster is indicated by red circle. **B** Violin plots of log-normalized, corrected count data showing expression of canonical excitatory neuron markers among the 12 Exc subtype snRNA-seq clusters. **C** Metascape analysis of enriched pathways among genes identified as upregulated in Exc5 compared to the other Exc clusters using the *FindMarkers* function of the Seurat R package. (i.) Network diagram indicating shared genes among enriched biological pathways. Node colors indicate clusters with similar functionality (indicated by cluster labels) and node sizes are proportional to the number of marker genes included in the pathway. Widths of lines linking pathway nodes are proportional to the number of shared genes between the two linked pathways. (ii.) The top 20 enriched biological pathway terms based on all upregulated Exc5 marker genes. Statistical significance (Log_10_ *p* value) is plotted on horizontal axes. Darker-colored bars indicated greater significance. Pathways are labeled with dots indicating membership in broad-functional based clusters based on clustering in i. **D** Feature plots of log-normalized, corrected count data showing expression of selected positive Exc marker genes with functions relating to major functional categories identified in panel C superimposed on UMAP plots. Darker violet coloring indicates stronger gene expression for corresponding nuclei. **E** Metascape analysis of enriched pathways among genes identified as downregulated in Exc5 compared to the other Exc clusters using the *FindMarkers* function of the Seurat R package. i and ii are formatted as in panel C for downregulated instead of upregulated Exc5 marker genes. **F** Feature plots of log-normalized, corrected count data showing expression selected negative Exc marker genes with functions relating to major functional categories identified in panel D superimposed on UMAP plots. Darker violet coloring indicated stronger gene expression for corresponding nuclei

The Exc5 cluster showed the most PD-upregulated DEGs and dysregulated biological pathways compared to other neuronal clusters in our dataset. Moreover, Exc5 was the only neuronal cluster showing a significant and robust overexpression of *SNCA* (Figs. 2C, S4B), further supporting a role for this neuronal subtype in later PD stages along the neuropathological progression of the disease [45, 46]. Thus, we sought to characterize in-depth this excitatory neuron subtype in the context of its relevance to PD (Fig. 3A). To this end, we first examined the expression of 10 canonical markers in all Exc and Inh neuronal clusters to annotate neuronal subtypes (Fig. S6). The vesicular glutamate transporter *SLC17A7* was expressed in all Exc subtypes (Fig. 3B, Fig. S6), indicating the predominance of glutamatergic neurons within our TC samples, and markers for GABA transmission (*SLC6A1*, *GAD1*) were expressed among Inh neuron clusters, as expected. Next, to further refine the characterization of the Exc neuronal subtypes and Exc5 in particular, we examined the expression of 56 marker genes previously used to categorize excitatory neuron subtypes [14, 47, 48] in nuclei of each of the Exc clusters (Fig. 3B, Fig S7). Exc5 cells showed high expression of *CBLN2*, *RASGRF2*, *CUX2*, and *PHACTR2*, but *RORB* or *FOXP2* expression was not detected (Fig. 3B). Four markers were highly expressed in Exc5, the autophagic genes *LAMP5* and *HPCAL1* [49–51]*,* and the long noncoding RNAs *LINC00507* and *LINC01500* (Fig. 2B). Exc1 was the only cluster showing a similar expression profile to Exc5 with relatively high co-expression of three out of the four markers, *LAMP5, HPCAL1* and *LINC00507*, whereas *LINC01500*, an upper cortical layer marker [47], was specifically expressed only in Exc5. Consistently with our findings, the expression of *LINC00507*, known to be involved in cortical development [52], was previously correlated with *LAMP5* expression [47]. Next, we further characterized the transcriptional profile of Exc5 cells compared to all other excitatory neuron clusters using FindMarkers in Seurat (Methods) and identified 432 upregulated and 498 downregulated marker genes (|log_2_FC| threshold of ≥ 0.25 and a gene-level FDR-adjusted *p* value threshold of < 0.01; Table S4). Metascape pathway enrichment analysis identified multiple enriched pathways for both upregulated and downregulated gene sets. Upregulated pathways (Fig. 3C) were related to neuronal development and organization, including the Roundabout (ROBO-SLIT) signaling pathway [53] and semaphorin genes (*e.g. SEMA3C;* Fig. 3D), both involved in axon guidance and neurite outgrowth [54]. Other upregulated pathways involved synapse formation, in particular the postsynaptic structure, and included *CBLN2*, involved in synaptic structure maintenance and glutamatergic synaptic transmission [55], and genes with roles in the postsynaptic structure such as glutamate receptor-encoding *GRIA4* (Fig. 3D). Additionally, intracellular ion homeostasis pathways were enriched with numerous genes, such as the voltage-gated calcium channel subunit-encoding *CACNA1E* (Fig. 3D)*.* Downregulated pathways (Fig. 3E) were enriched for genes involved in presynaptic organization (e.g., Synaptoporin-encoding *SYNPR*), synaptic transmission (e.g., voltage-gated potassium channel subunit-encoding *KCNH5*), inhibitory signal reception (e.g. GABA receptor subunit-encoding *GABRG3*), and calcium-response (e.g., *CDH6* and *PDE1C*; Fig. 3F).

### Cell type and subtype-specific differential chromatin accessibility in PD

**Fig. 4:**
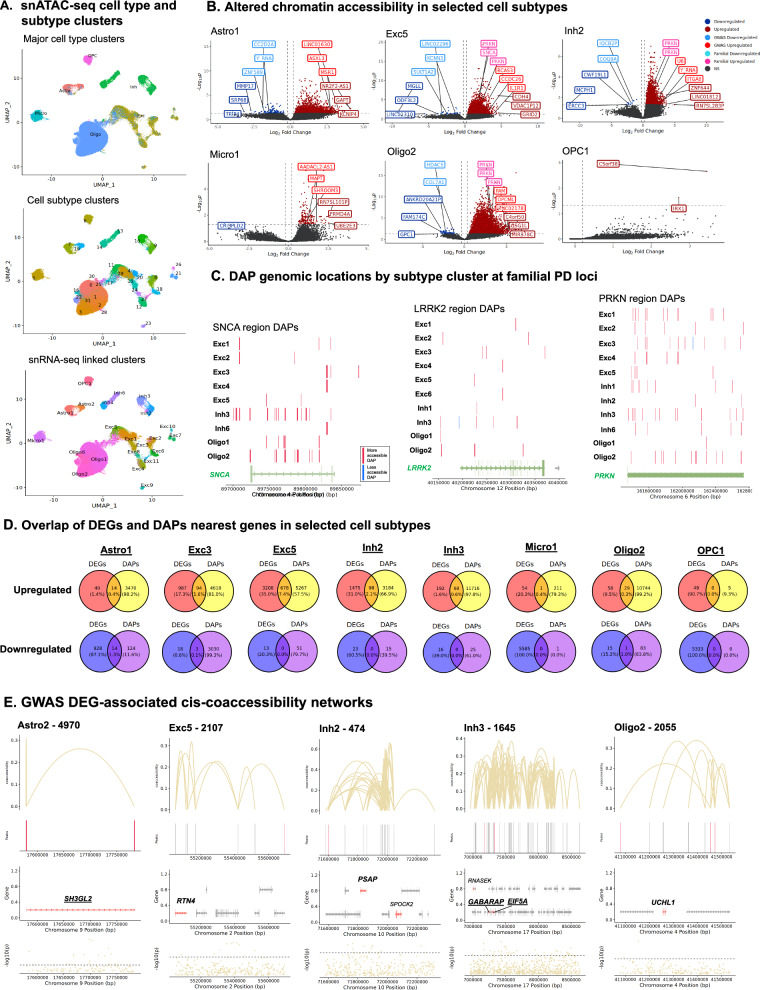
Differential chromatin peak accessibility in snATAC-seq cell subtype clusters. **A** UMAP dimensional reduction plots of snATAC-seq nuclei data indicating major cell types (left panel), subtype clusters based on snATAC-seq data (center panel) and snRNA-seq-linked clusters determined via label transfer (right panel). **B** Unbiased volcano plots for example linked-subtype clusters belonging to each of the major cell types. Log_2_ fold change (FC) in peak accessibility between PD and normal control samples is plotted against –log_10_ *p* value (FDR). Points representing DAPs with statistically significant (*p* < 0.05) upregulation in PD are shown in dark red while DAPs with significant downregulation are shown in dark blue. Peaks without significantly differential expression are shown as gray points. The three DAPs with the highest absolute fold change (|log_2_FC| > 0.2) in the more- and less-accessible categories are labeled with their nearest genes in dark red and dark blue, respectively. The closest genes to the top three more- and less-accessible DAPs within 500 kb of PD GWAS-SNVs are labeled in teal and pink, respectively. The DAPs closest to familial PD genes with the highest absolute log_2_FC in the more- and less-accessible categories are labeled in pink and teal, respectively. **C** Genome track plots showing the genomic locations of DAPs in relation to the hereditary PD genes *SNCA*, *LRRK2*, and *PRKN* for each of the indicated linked cell subtypes. More accessible DAPs are shown in red while less accessible DAPs are shown in blue. Gene coding regions are shown in green, with direction of transcription indicated by arrows, and exons indicated by vertical bars. The remaining area of the coding region comprises the gene introns. **D** Venn diagrams showing numbers of overlapping DEGs and DAP closest genes for upregulated DEGs/more-accessible DAPs (red/yellow), and downregulated DEGS/less-accessible DAPs (blue/violet) for selected clusters. **E** Diagrams of cis-coaccessibility networks overlapping PD GWAS-SNV regions. Vertical lines at the top of each diagram indicate chromatin peaks and Bezier curves indicate coaccessibility of distal chromatin peaks to peaks overlapping the promoters or intron 1 regions of DEGs. DAPs with greater accessibility in PD are shown in salmon while DAPs with reduced accessibility in PD are shown in blue. Non-differentially-accessible peaks are shown in grey. Below the peak linkage plots, gene exons overlapping CCAN regions are depicted as arrows indicating the directionality of transcription. DEGs are labeled with gene names, and names of genes with distal peak coaccessibility in promoter or intron 1 regions are labeled in bold text. PD GWAS-DEGs are labeled with underlined text. PD-upregulated DEGs are shown in salmon, downregulated DEGs are shown in blue, and non-differentially expressed genes are shown in grey. At the bottom of each diagram, a Manhattan plot is shown indicating -log_10_ of *p*-values for PD-association of chromosome loci within CCAN region as reported by Nalls et al. [5]. Dotted lines in Manhattan plots indicate statistical significance threshold (*p* = 0.05). GWAS-identified PD-associated SNVs are indicated via red dots and labeled on Manhattan plots

In the snATAC-seq dataset, 167,445 nuclei were retained after QC filtering (Table S5) and Louvain community detection distinguished 32 cell subtype clusters representing the same six major cell types as in the snRNA-seq data (Fig. 4A). We also checked for donor-based batch effects in snATAC-seq clustering by examining the distribution of nuclei across subtype clusters for each donor sample, and showed again qualitatively even distribution of donor samples and diagnosis groups across subtypes (Fig. S9). Comparisons of major cell types and subtype clusters using MASC did not reveal significant depletion or enrichment of any cell types in PD compared to control, consistent with the snRNA-seq findings and as expected from the observed TC McKeith stages (Fig. S3B). We further mapped the 32 snATAC-seq clusters to 22 of the corresponding snRNA-seq clusters (Fig. 4A), and focused the downstream analyses on these corresponding cell subtype clusters. Chromatin accessibility differential analysis between PD and control groups by NEBULA (number of nuclei, age, sex, and PMI were included as covariates in the model) identified differentially accessible peaks (DAPs) in 20 of the subtype clusters (FDR < 0.05 and a log_2_ FC cutoff of > 0.2; Figs. 4B and S10). Of these 20 clusters, Oligo2 demonstrated the highest number of DAPs with 28,377, while OPC1 had the fewest with only 5 DAPs (Figs. 4B and S10). All clusters showed higher numbers of DAPs with increased as opposed to decreased accessibility in PD (Fig. S10).

The snRNA-seq and snATAC-seq assays were performed in parallel by splitting and simultaneous processing of one nuclei preparation for each donor sample to generate corresponding gene expression and chromatin accessibility libraries, respectively, for each study subject. This parallel processing minimized technical variables and variability in cell type composition between the assays, thereby facilitating downstream integrative multi-omics analyses to better understand the relationships between DEGs and DAPs. More open DAPs were identified within coding and promoter regions of three fPD genes: *SNCA*, *LRRK2*, and *PRKN,* in several cell subtypes (Fig. 4C). Overall, correlation between DEGs and DAPs, annotated by the closest gene, was fairly low, with relatively few DEGs having corresponding DAPs with the same directionality (upregulated/more accessible or downregulated/less accessible) within the same cell subtype (Fig. 4D). Notably, the Exc5 cluster had the highest number of more accessible DAPs overlapping with upregulated DEGs (n = 678), followed by Inh2 and Exc3 (n = 98 and 94, respectively). These results suggest that in many cases open chromatin sites may not affect the closest gene, but may instead be linked to more distal genes.

### Mechanisms of gene dysregulation in PD

#### Changes in chromatin accessibility of candidate *cis*-regulatory elements (cCREs)

To evaluate the effect of PD-dependent changes in chromatin accessibility on gene dysregulation in PD, we first employed Cicero [56] to identify coaccessible peaks and construct *cis*-coaccessibility networks (CCANs) using the snATAC-seq data of the PD nuclei. We applied a threshold coaccessibility score of ≥ 0.2 [56, 57] between each peak and at least one other peak in the network and revealed a total of 29,968 CCANs across all 22 clusters (Table S6). Next, within each CCAN we catalogued distal peaks that were coaccessible to proximal peaks overlapping the promoters or intron 1 of genes and the resulting peak pairs defined cCREs and their linked genes. The downstream analysis was confined to a subset of CCANs that encompassed both DAPs and cCREs linked to DEGs with the same directionality (unidirectional CCANs). The analysis identified 115 unidirectional CCANs (Table S6), including 2 CCANs overlapping PD GWAS-DEGs (Fig. 4E). Examples of cCRE-linked DEGs within unidirectional CCANs included *RTN4* in Exc5, encoding an inhibitor of neurite outgrowth [58], *GABARAP* in Inh3*,* encoding an autophagy-associated GABA receptor [59], and the proteasome-associated *UCHL1* in Oligo2, involved in *SNCA* degradation [60] (Fig. 4E).

#### Changes in transcription factor (TF) abundance

**Fig. 5:**
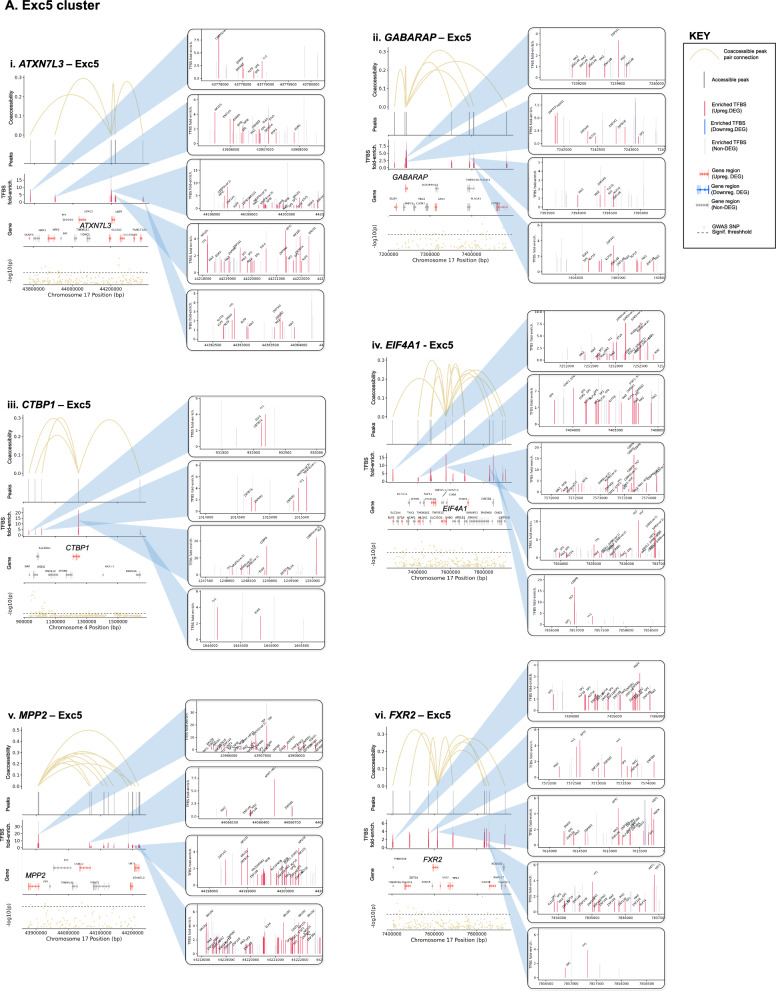

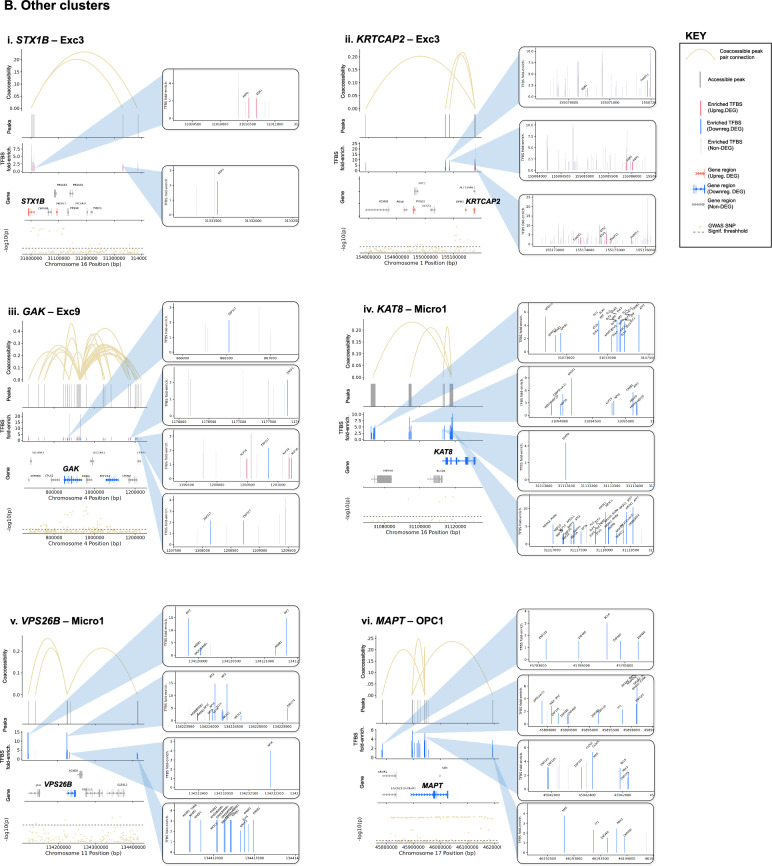

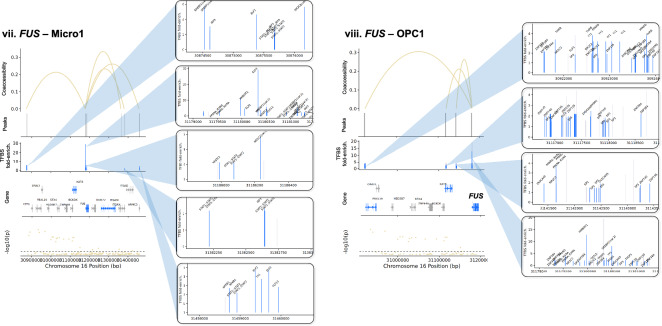
TFBS motif enrichment within regulatory regions and candidate cis-regulatory elements (cCREs) of PD GWAS DEGs. **A**, **B** Diagrams of candidate cis-regulatory elements (cCREs) of example PD GWAS-DEGs and associated enriched transcription factor binding motif sites (TFBS). Vertical lines at the top of each diagram indicate chromatin peaks and Bezier curves indicate coaccessibility of distal chromatin peaks to peaks overlapping the promoters or intron 1 regions of PD GWAS-DEGs. Non-differentially-accessible peaks are shown in grey. Below the peak linkage plots, gene exons overlapping cCRE regions are depicted as arrows indicating the directionality of transcription. DEGs are labeled with gene names, and names of genes with distal peak coaccessibility are labeled in enlarged text. PD-upregulated DEGs are shown in salmon, downregulated DEGs are shown in blue, and non-differentially expressed genes are shown in grey. At the bottom of each diagram, a Manhattan plot is shown indicating −log_10_ of *p* values for PD-association of chromosome loci within cCRE region as reported by Nalls et al. [5]. Dotted lines in Manhattan plots indicate statistical significance threshold (*p* = 0.05). GWAS-identified PD-associated SNPs are indicated via red dots and labeled on Manhattan plots. Boxes to the right of cCRE diagrams depict enlarged regions of chromatin peaks indicated by blue triangles and show the locations of enriched TFBS represented by vertical lines. Motifs for TF-DEGs upregulated in PD are shown in red, while those for downregulated TFs are shown in blue. Motifs for non-DEG TFs are shown in grey. Heights of TFBS bars indicate the fold-enrichment of binding motifs for the associated TF within the cCRE peaks compared to elsewhere in the genome. **A** cCREs of the Exc5 cluster. **B** cCREs of additional clusters

Additionally, gene dysregulation in PD may result from altered TF expression. Thus, we first identified a total of 142 cCREs linked to 104 unique PD GWAS-DEGs (out of 628 total PD GWAS-DEGs examined) across 18 clusters (Fig. 5; Table S7). We then predicted TF binding sites within the promoter/intron 1 of these PD GWAS-DEGs and their linked cCREs in each cell subtype for TFs expressed in ≥ 10% of cells using HOMER [61]. The analysis revealed enrichment of TF binding sites (TFBS) within 125 cCREs linked to a total of 90 unique PD GWAS-DEGs across 17 out of the 18 analyzed cell subtypes (fold-enrichment cutoff ≥ 1.2 and FDR < 0.05; Fig. 5, Table S8). Notably, 93 of the cCREs with TFBS enrichment showed at least one TF that was also encoded by a PD-associated DEG (hereafter TF-DEG). Notably, Exc5 showed the highest number of cCRE-linked upregulated PD GWAS-DEGs, and the strongest enrichment for TF-DEGs (Fig. 5A, Table S8). For downregulated PD GWAS-DEGs, the strongest enrichment for TF-DEGs was found in Micro1 and OPC1 (Fig. 5Biv-vi, Table S8). Enrichment for binding sites of TF-DEGs in cCREs linked to upregulated and downregulated PD GWAS-DEGs was also identified in other cell subtype clusters (Fig. 5Bi-iii, Table S8).

**Fig. 6:**
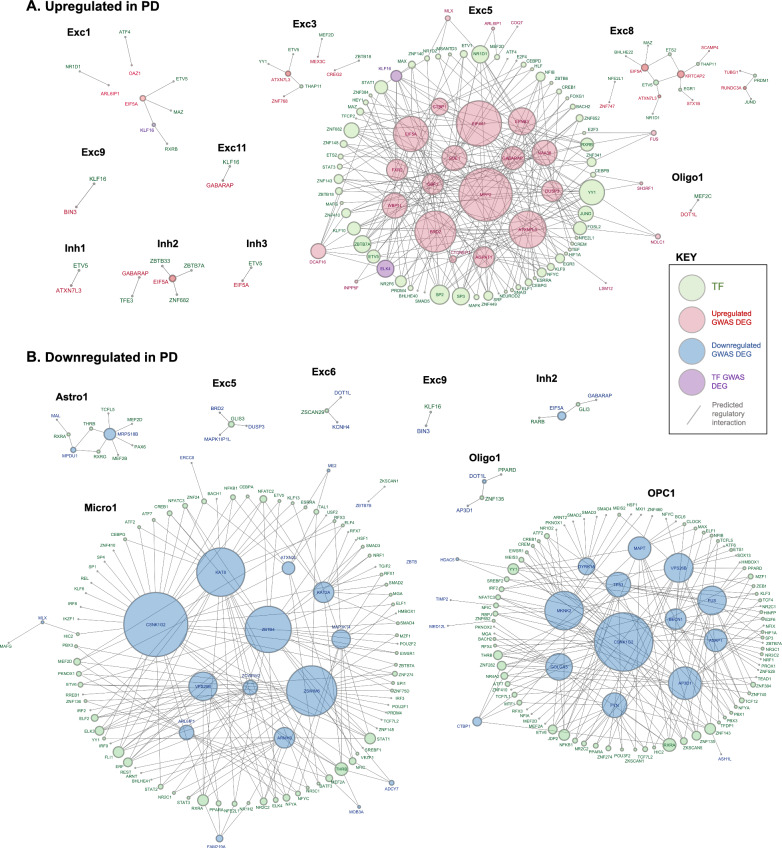
Predicted transcription factor regulatory networks for PD GWAS-DEGs. **A**, **B** Diagrams of predicted regulatory networks of PD GWAS-DEGs and the TF-DEGs predicted to regulate them based on enrichment of TF binding motif sites (TFBS) within the promoters and intron 1 sequences and distal coaccessible peaks of the associated PD GWAS-DEGs, generated by Cytoscape software. Network diagrams are shown for each linked snRNA-seq/snATAC-seq cluster in which enriched TFBS were identified for TF-DEGs within cCRE region of GWAS DEGs. Red nodes on diagrams represent PD-upregulated GWAS DEGs, blue nodes represent PD-downregulated GWAS DEGs, green nodes represent TF-DEGs, and purple nodes represent PD GWAS-DEGs that are also TF-DEGs. Lines linking nodes indicate predicted regulatory relationships between TF-DEGs and PD GWAS-DEGs based on TFBS enrichment within cCRE regions. Node sizes are proportional to the number of linkages predicted (Number of PD GWAS-DEGs predicted to be regulated by a particular TF or number of TFs predicted to regulate a particular PD GWAS-DEG). Panel A depicts PD GWAS-DEGs and TF-DEGs upregulated in PD, while panel B depicts those downregulated in PD (directionality of regulation is always the same between TF-DEG and PD GWAS-DEG)

In order to identify TFs potentially acting as master regulators of PD-associated gene expression, we examined networks of TF-DEGs with TFBS enrichment in cCRE regulatory regions of target PD GWAS-DEGs for each cell subtype cluster (Fig. 6). Among upregulated PD GWAS-DEGs, the Exc5 cluster produced the most extensive network, comprising 63 TF-DEGs and 27 linked target DEGs (Fig. 6A). Among these TFs, several emerged as candidate regulators of larger numbers of PD GWAS-DEGs, with YY1 enriched in cCREs of the largest number of target DEGs (n = 13). Notably, ELK4 and KLF16 (9 and 6 linked-DEGs, respectively) were also themselves encoded by upregulated PD GWAS-DEGs in the Exc5 cluster. Among the targeted DEGs, postsynaptic scaffold protein-encoding *MPP2* [62] was predicted to be regulated by the highest number of TF-DEGs (n = 27; Fig. 6A, Table S8).

For the downregulated PD GWAS-DEGs, the largest TF-DEG/PD GWAS-DEG regulatory networks were identified in the Micro1 cluster, with 79 TF-DEGs predicted to regulate 18 PD GWAS-DEGs, and the OPC1 cluster, with 87 TFs predicted to regulate 17 PD GWAS-DEGs (Fig. 6B). However, compared to the Exc5 cluster network, there were fewer TFs in the Micro1 and OPC1 clusters predicted to regulate high numbers of GWAS-DEGs.

#### PD-correlated genetic variants altering TF binding affinity

Alterations in binding affinities of TFs to cCREs and regulatory regions may also contribute to dysregulation of gene expression in PD. To asses this mechanism we used MotifbreakR [63] to predict the effects of genomic variation [64] on TF binding affinity within sequences of cCREs and promoter/intron 1 regions linked to 41 selected PD GWAS-DEGs in corresponding cell subtypes. We identified a total of 1175 unique variants predicted to impact binding of a total of 599 TFs, including 1035 SNVs, 27 insertions, and 113 deletions (Table S9). TFs showing changes in the affinity of their binding sites also included TF-DEGs, with the highest number (n = 24) identified in the Exc5 cluster (Table S10). Some of these TF-DEGs (including YY1) were predicted to regulate multiple PD GWAS-DEGs (described above).

**Fig. 7:**
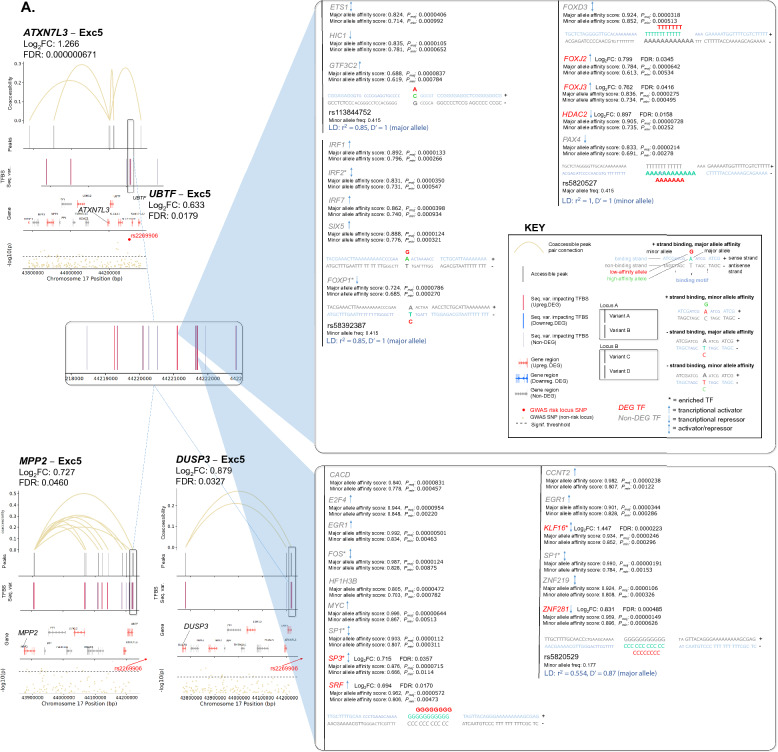

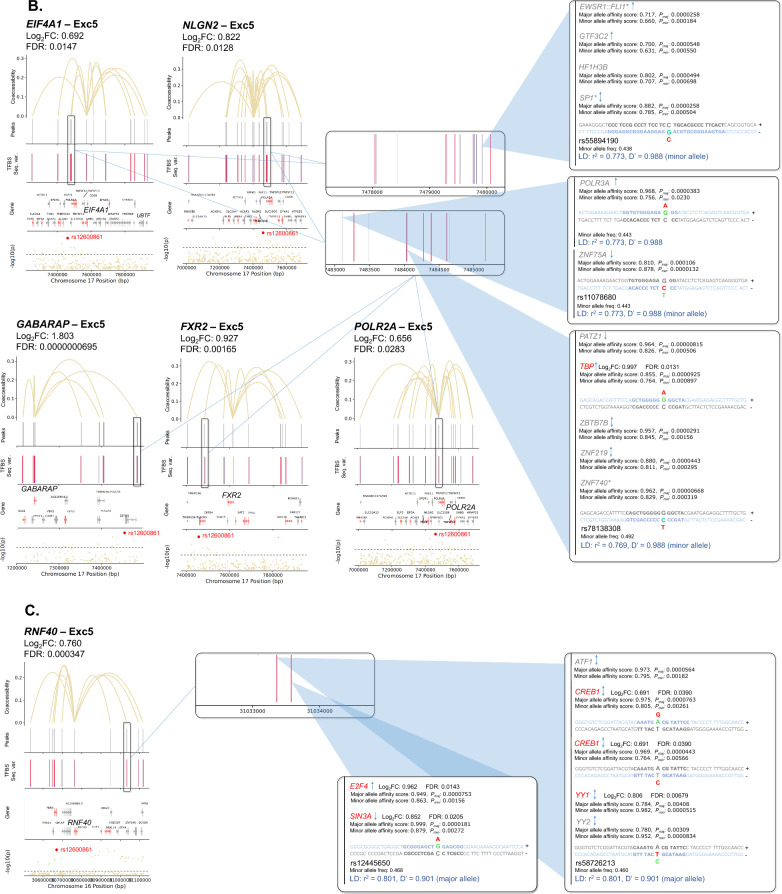

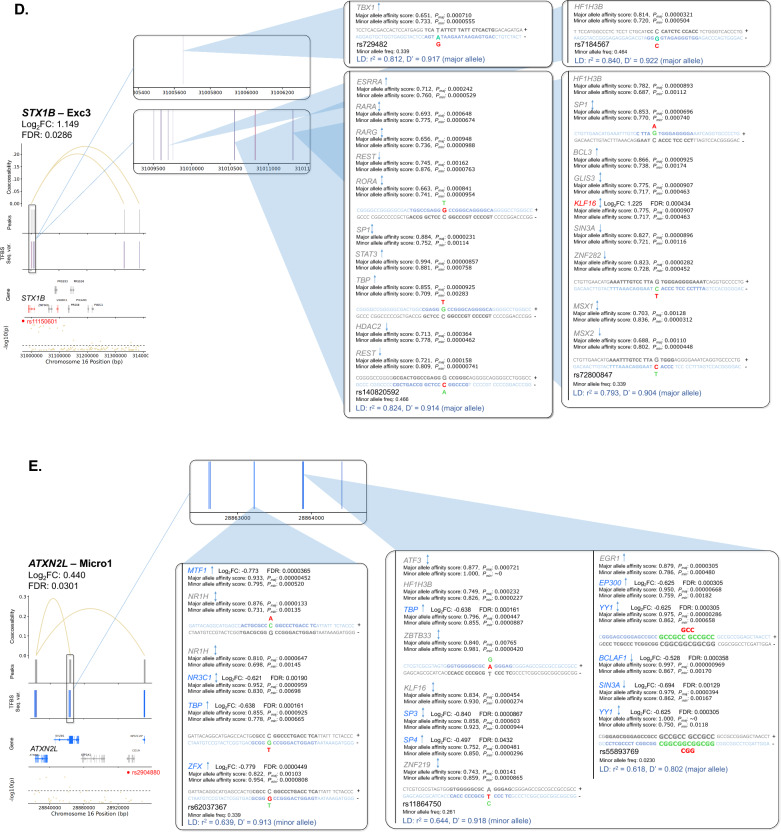
Sequence variants predicted to impact binding affinity for TFs in regulatory elements of PD GWAS-DEGs. **A**–**E** Diagrams of cCREs of example PD GWAS-DEGs and associated TFBS. PD-versus-normal fold change and associated FDR values for the relevant PD GWAS-DEGs are shown for each diagram. Vertical lines at the top of each diagram indicate chromatin peaks and Bezier curves indicate coaccessibility of distal chromatin peaks to peaks overlapping the promoters or intron 1 regions of PD GWAS-DEGs. Non-differentially-accessible peaks are shown in grey. Below the peak linkage plots, gene exons overlapping genomic regions are depicted as arrows indicating the directionality of transcription. DEGs are labeled with gene names, and names of genes with distal peak coaccessibility are labeled in enlarged text. PD-upregulated DEGs are shown in salmon, downregulated DEGs are shown in blue, and non-differentially expressed genes are shown in grey. At the bottom of each diagram, a Manhattan plot is shown indicating −log_10_ of *p* values for PD-association of chromosome loci within genomic region as reported by Nalls et al. [5]. Dotted lines in Manhattan plots indicate statistical significance threshold (*p* = 0.05). GWAS-identified PD-risk-associated SNVs are indicated via red dots and labeled on Manhattan plots. Boxes to the right of cCRE diagrams depict enlarged regions of chromatin peaks connected by blue lines and vertical lines show the locations of sequence variants predicted to impact binding affinity of TFs for associated TFBS. Variants in motifs for TF-DEGs upregulated in PD are shown in red, while those for downregulated TF-DEGs are shown in blue. Motif variants for non-DEG TFs are shown in grey. Sequence tracks to the right of sequence variant plots depict the specific sequence variations predicted to affect binding affinity of the indicated TF. The DNA strand containing the TFBS is shown in color, while the opposite strand is shown in greyscale. Binding motifs are indicated by bold lettering and shown in dark blue on the binding strand. Sequence variants are shown in enlarged letters within motif regions, with the major variant shown aligned with the chromosomal sequence and the minor variant placed externally. Variant nucleotides associated with greater predicted binding affinity are shown in green, while those associated with lower affinity are shown in red. Above the sequence tracks, gene names of TFs with predicted binding affinity changes from associated sequence variants are shown. Below these, the major and minor allele binding affinity scores, and the major and minor allele *p* values are indicated in black lettering. Upregulated TF-DEG names are shown in red, and downregulated TF-DEG names are shown in blue. The fold change and associated FDR values for the DEG TFs are shown to the right of the gene names. Non-DEG TF names are shown in grey. Asterisks next to TF names indicate TFBS enriched within the cCRE peaks indicated compared to elsewhere in the genome. Up arrows indicate that the TF is a known or predicted transcriptional activator; down arrows indicate a known or predicted transcriptional repressor; bi-directional arrows indicate both transcriptional activation and repression; absence of arrow indicates no information available. Below the sequence tracks, sequence variant IDs are indicated, and r^2^ and D′ linkage disequilibrium values of sequence variant loci with the most proximal PD GWAS-SNV are indicated in blue. Whether the major or minor allele of the variant correlates with the PD risk variant of the GWAS-SNV is also noted in parentheses

Next, we sought insight into the relationships between the identified regulatory variants and PD risk loci. We examined the linkage disequilibrium (LD) between each candidate regulatory variant and the nearest PD GWAS-SNV using both r^2^ and D′ metrics. Out of the 1175 candidate regulatory variants, 19 were in LD with a PD GWAS-SNV (r^2^ and D′ ≥ 0.5; Table S11). Several variants were predicted to impact expression of multiple PD GWAS-DEGs, particularly in the Exc5 cluster. A prominent example involved the PD GWAS-DEGs *UBTF*, *ATXN7L3*, *MPP2*, and *DUSP3* (Fig. 7A, Table S11). Two of the TFs with predicted altered binding affinity in cCREs of these genes, IRF2 and FOXP1, also exhibited TFBS enrichment in cCREs of *ATXN7L3* and *MPP2*, respectively. The minor allele of deletion variant rs5820527 was found to have perfect LD with the PD risk allele of PD GWAS-SNV rs2269906 (r^2^ = 1, D′ = 1), suggesting that disrupted binding of associated TFs (FOXD3, PAX4, and the TF-DEGs: FOXJ2, FOXJ3, and HDAC2) may contribute mechanistically to PD risk. The major allele of another deletion variant in this region (rs5820529) was in high LD with the same PD GWAS-SNV (r^2^ = 0.554, D′ = 0.87; Fig. 7A, Table S11). Noteworthily, TFs SP3 and KLF16 are predicted to have enhanced binding to this PD-risk allele and are encoded by upregulated DEGs in the Exc5 cluster. Furthermore, SP3 binding motifs are enriched within cCRE regions of both *ATXN7L3* and *MPP2*, while KLF16 is enriched for binding in cCRE regions of *MPP2*. Both are also among the overall most enriched TFs within cCREs of PD GWAS-DEGs in Exc5, and KLF16 is itself also encoded by a PD GWAS-DEG (Fig. 6A). Altogether, these observations suggest that transcriptional upregulation of PD GWAS-DEGs such as *ATXN7L3* and *MPP2* by the TFs SP3 and KLF16 may increase PD risk. FOS1 and SP1 are also enriched for binding within *MPP2* cCRE regions and were predicted to have enhanced binding to this PD risk-associated allele, as was ZNF219, an important regulator of *SNCA* expression [65].

A second example of PD risk variants potentially impacting regulation of multiple Exc5 PD GWAS-DEGs was discovered on chromosome 17. cCREs of 5 PD GWAS-DEGs (*EIF4A1*, *NLGN2*, *GABARAP*, *FXR2*, and *POLR2A*) had predicted differential binding at 3 variant loci with high LD to PD GWAS-SNV rs12600861 (Fig. 7B, Table S11). SP1 binding motifs were enriched in cCREs of both *EIF4A1* and *GABARAP*, and the binding affinity of this TF was predicted to be disrupted by the minor allele of regulatory SNV rs55894190 (r^2^ = 0.773, D′ = 0.988 with PD risk allele of PD GWAS-SNV rs12600861) within the *EIF4A1* and *NLGN2* cCREs. The minor allele of a second SNV in this region (rs78138308) was also in high LD with the same PD risk allele (r^2^ = 0.769, D′ = 0.988). Binding of TF-DEG TBP was predicted to be disrupted by this variant, as was that of ZNF40, enriched in cCREs of *EIF4A1*, *GABARAP*, *FXR2*, and *POLR2A*, and *ZNF219*.

On chromosome 16, two SNVs (rs12445650, rs58726213) showed strong LD with PD GWAS-SNV rs12600861 (r^2^ = 0.801, D′ = 0.901) within cCRE peaks linked to the PD GWAS-DEG *RNF40* in Exc5 nuclei (Fig. 7C, Table S11). The risk allele of PD GWAS-SNV rs58726213l was in high LD with the major allele of regulatory SNV rs58726213, which was predicted to have a stronger binding affinity of cAMP response element-binding TFs ATF1 and CREB1, the latter of which was also encoded by an upregulated DEG in Exc5. These findings suggest the possible involvement of calcium-responsive pathways in to *RNF40* regulation. Moreover, binding of the TF-DEG YY1 was predicted to be disrupted by the PD risk variant allele at this locus.

Sequence variants in high LD with PD risk alleles were also identified in other cell subtype clusters, including in cCREs of upregulated PD GWAS-DEGs in neuronal cluster Exc3 (Fig. 7D, Table S11), and downregulated PD GWAS-DEGs in microglial cluster Micro1 (Fig. 7E, Table S11), as well as numerous other subtype clusters (Table S11).

## Discussion

In this study we analyzed snRNA-seq and snATAC-seq data *in parallel* from TC samples of the same donors to examine the transcriptomic and epigenomic landscapes associated with PD progression in cell subtypes (Fig. 1). Our study design incorporated innovative approaches with regard to both biology and analytical methodology in order to uncover novel genetic factors underlying PD. Biologically, the analysis of the TC, most strongly affected during later stages of the disease, provided new insights into the molecular changes leading to PD progression. Moreover, the TC regions of our study subjects were only mildly affected, thus allowing us to capture molecular changes occurring prior to neurodegenerative processes. Furthermore, while a recent study reported PD-associated transcriptomic and chromatin accessibility changes at a cell type-specific level [18], to the best of our knowledge our study is the first comprehensive multi-omics single cell analysis of PD at a granular cell subtype level of precision. Analytically, this is the first study that has examined several classes of variants, including short structural variants (SSVs) such as indels, in relation to mechanisms of transcriptional dysregulation in PD.

We characterized a specific subpopulation of vulnerable cortical glutamatergic neurons with strikingly altered gene expression in PD. The high number of DEGs identified for the Exc5 cluster was of particular interest given the important role of excitatory neurons in driving PD pathology [13–15]. Furthermore, the Exc5 subtype of glutamatergic neurons was the only neuronal cell subtype showing increased *SNCA* expression. SNCA protein is the main component of Lewy bodies and is the pathological hallmark of PD [66], and overexpression of *SNCA* has been implicated in PD pathogenesis (we reviewed previously [45]). *SNCA* mutations were also identified in rare families with fPD [4, 67]. Thus, upregulation of *SNCA* levels further supports the important role of Exc5 in PD. The signature of the Exc5 subtype was similar to Exc1 in marker gene expression yet the former population expressed > sevenfold more DEGs. The genetic markers defining Exc5 suggested that compared to other excitatory neuron subtypes Exc5 cells were characterized by dysregulation of several key neuronal functions including: (1) cell development and organization, guided in part by the Roundabout signaling pathway; (2) synaptic organization, prioritizing postsynaptic reception of excitatory neurotransmitters and downregulation of inhibitory receptors and genes involved in presynaptic signal transmission; and (3) regulation of intracellular calcium ion levels with comparative repression of downstream calcium response elements. Neuronal organization and axon guidance have been previously implicated in PD vulnerability [68] as increased axonal arborization has been associated with increased reactive oxygen species (ROS) stress due to mitochondrial hyperactivity, while semaphorin-mediated reduction of arborization led to decreased ROS production and increased neuronal resilience [69]. We suggest that specific organizational and synaptic structural features of Exc5 neurons may contribute to the disproportionate transcriptional dysregulation in PD observed for this cell type. Further investigations in PD model systems are needed to elucidate the specific biological functions of the neuronal subtype represented by Exc5, and to better understand its role in PD pathology.

Additionally, using our integrative single cell multi-omic approach, we examined several different regulatory mechanisms potentially governing transcriptional dysregulation in the context of PD pathogenesis and disease progression. First, we identified PD-associated changes in chromatin accessibility within cCREs coaccessible to the promoter/intron 1 regions of DEGs which may thereby have a mechanistic effect on dysregulation of these DEGs in the PD state. Next, we identified enrichment for binding motifs of differentially expressed TFs within DEG-linked cCREs, indicating that these TFs may act as master regulators of numerous genes driving PD neuropathological progression. These results suggested that changes in the expression levels of a relatively small number of TFs could mechanistically drive large-scale dysregulation of gene expression within key disease-relevant cell subtypes such as Exc5. Finally, we catalogued regulatory genetic variants, both SNVs and SSVs, that are predicted to change the affinities of TF binding sites within cCREs linked to PD GWAS-DEGs, and identified several regulatory SNVs and SSVs that were in strong LD with PD GWAS-SNVs. Our finding suggested that transcriptional regulatory variants underlie mechanistically, at least in part, the disease risk impact of PD GWAS loci.

Our analysis pointed at YY1, SP3, and KLF16 as candidate master regulators of gene expression in PD, especially within the Exc5 cell subtype. All of these TFs were also found to be differentially expressed in PD, and their binding affinities within cCREs were impacted by regulatory variants showing high LD with GWAS SNVs. YY1 can act as both a transcriptional activator and repressor, and is thought to play an important role in neuronal development and function [70, 71]. YY1 has not been previously associated with PD, but has been suggested to play a role in Alzheimer’s neurodegeneration by regulation of BACE1 and other proteins involved in amyloid-beta (Aβ) generation [70], and is also known to mediate DNA repair [72]. SP3 is involved in many developmental processes [73], as well as in inflammatory NF-κB signaling [74]. The gene encoding KLF16 was also mapped within a PD-GWAS region and this TF functions both in transcriptional activation and repression [75]. KLF16 has also been shown to inhibit neurite outgrowth [76], and to regulate dopaminergic synaptic transmission in the striatum [77]. Binding motifs for KLF16 were enriched within cCRE regions of the postsynaptic scaffold gene *MPP2*, and KLF16 binding in this region was predicted to be enhanced by an identified PD risk-associated sequence variant. Of note, MPP2 plays an essential role in synaptic ion channel function and glutamatergic neuron excitability [78], thus changes in its expression may result in disrupted postsynaptic structure and neurotransmission. Consistently, post-synapse-associated genes were among the most highly enriched markers for the Exc5 neuronal subtype.

Our study presents important advancements to the field of PD genetics. However, there are several limitations. First, while the TC region has previously been shown to be involved in later stages of PD [79–84], in this work, all PD donor samples were in stages preliminary to major disease progression to the cortical brain region, and the majority of samples thus exhibited little to no Lewy pathology within the TC, based on established metrics [85]. Furthermore we did not find any dopaminergic neuron clusters within our dataset, the subtype most highly impacted by neurodegeneration in early PD stages. Thus, our data reflect PD-associated transcriptional changes in intact TC tissue preceding major neurodegenerative effects in this region, providing insight into preliminary genetic mechanisms leading to disease progression and subsequent neurodegeneration and cognitive effects. As our interest focused on disease progression into tissues affected in later stages, however, the donor samples did show pronounced pathology within early-affected tissues such as the SN. Understanding the effects of the disease in later stages may aid in the development of treatment strategies to halt disease progression. Another limitation stems from the uncertainty inherent in linking cell subtype clusters derived from parallel datasets. We previously estimated the average accuracy of such cluster linking to be approximately 75.6%, and found that this level of certainty was sufficient to provide meaningful insight into the relationships between epigenetic and transcriptomic observations [57]. The recent development of simultaneous multi-omic single-nucleus sequencing methods [86] would overcome this limitation. Additionally, we employed covariate selection analysis and tailored our statistical models to control for identified confounding variables as well as for random effects associated with specific donor samples, but it is not possible to completely control for all possible confounding effects when dealing with complex biological systems. Moreover, while we provided an expanded investigation into the impact of genomic variation on gene expression in PD by including both SNVs and indels in our analysis, our methodology using *MotifbreakR* did not include the assessment of longer repeat structural variants. Finally, we reiterate the caveat that the mechanistic relationships described here between chromatin accessibility, sequence variation, and gene expression are predictive in nature and empirical evidence obtained through controlled experimentation will be required to validate these relationships and their roles in PD. Physical interactions between cCREs and putative target genes identified in silico may be validated using novel single-cell methods such as Promoter Capture Hi-C for 3D genomic mapping [87].

Here, we have provided mechanistic insights into the gene expression patterns associated with PD. Examining TC tissues with minimal neuropathological perturbations opened a window into transcriptional dysregulation occurring in early stages of PD progression to the cortical brain, prior to neurodegeneration in this region. Thus, while these changes in gene expression occur subsequently to disease onset in earlier-affected tissues, they likely represent molecular events specifically driving the progression of PD into the cortex. Collectively, we described PD-associated changes in the cross talk between cCREs, TFs, genetic variants, and gene expression. Furthermore, here for the first time, we identified candidate PD regulatory variants that are in high LD with PD GWAS-SNVs and hence are candidate PD causal variants. Follow up experimental validation in PD model systems is warranted to determine the effects of these candidate regulatory variants on TF binding and gene expression and to confirm their relevance to PD. Together, these findings provide new insight into the underlying mechanisms of transcriptional dysregulation in PD at a cell subtype-specific level and further support the role of gene dysregulation in PD risk and progression.

## Methods

### Human post-mortem brain tissue samples

Frozen human TC tissue samples from donors clinically diagnosed with PD (*n* = 12) were obtained from the Banner Sun Health Research Institute (BSHRI) [88], and neurologically healthy control donor samples (Normal) (*n* = 12) were obtained from the Kathleen Price Bryan Brain Bank (KPBBB) at Duke University. Unaffected controls were derived from donors with no clinical history of neurological disorder and samples had no neuropathological evidence of neurodegenerative diseases. Donor patient PD diagnoses were defined by the presence of two of the three cardinal clinical signs of resting tremor, muscular rigidity and bradykinesia. Additionally, diagnoses of all 12 PD samples were confirmed in autopsy by observation of pigmented neuron loss and the presence of Lewy bodies in the SN. Neuropathological states of PD samples were confirmed *postmortem* using established clinical practice recommendations for McKeith scoring [85] and staging via the Unified Staging System for Lewy Body Disorders (USSLB) [89]. All PD samples for which information was available had McKeith scores ranging from moderate to severe (2–4) in both the amygdala and SN. Where available, TC McKeith scores for most of the PD samples were either 0–1, with one sample each receiving scores of 2 and 3, indicating mild or absent PD pathology in this region for the majority of samples. USSLB stages of PD samples ranged from II–IV. PD samples 96-36 and 96-49 were lacking specific USSLB stage determination due to harvesting prior to BSHRI standardization of stage determination protocol. All tissue donors were of European ancestry. The demographics and other metadata for this cohort are detailed in Tables 1 and S1. Complete pathology analysis results of PD samples are provided in Table S12. The project was approved for exemption by the Duke University Health System Institutional Review Board. The methods described were conducted in accordance with the relevant guidelines and regulations.

### Cohort statistics

For comparisons of demographic variables, R statistical programming language was used. Age and post-mortem interval (PMI) of female PD was compared to female Normal, and age and PMI of male PD was compared to male Normal (Table S13). The Shapiro–Wilk test was used for normality, Bartlett’s test for equal variance of normally distributed data, and Levene’s test for equal variance of non-normally distributed data. If compared groups were normally distributed but had unequal variance, two sample Welch’s *t*-tests were used to determine differences between group means. If groups were not normal but had equal variances, a Mann–Whitney’s *U* test was run.

### Nuclei isolation from post-mortem human brain tissue

The nuclei isolation procedure has been described [57], and was based on previous studies [90, 91] and optimized for single-cell experiments. 100–200 mg of human TC brain tissue samples were thawed in Lysis Buffer (0.32 M Sucrose, 5 mM CaCl_2_, 3 mM Magnesium Acetate, 0.1 mM EDTA, 10 mM Tris–HCl pH 8, 1 mM DTT, 0.1% Triton X-100) and homogenized with a 7 ml dounce tissue homogenizer (Corning) and filtered through a 100 μm cell strainer, transferred to a 14 × 89 mm polypropylene ultracentrifuge tube, and underlain with sucrose solution (1.8 M Sucrose, 3 mM Magnesium Acetate, 1 mM DTT, 10 mM Tris–HCl, pH 8). Nuclei were separated by ultracentrifugation for 15 min at 4 °C at 107,000 RCF. Supernatant was aspirated, and nuclei were washed with 1 ml Nuclei Wash Buffer (10 mM Tris–HCl pH 8, 10 mM NaCl, 3 mM MgCl_2_, 0.1% Tween-20, 1% BSA, 0.2 U/μL RNase Inhibitor). 800 μL resuspended nuclei were transferred to a microcentrifuge tube designated for the 10X Genomics single-cell ATAC assay while 200 μL was transferred to a microcentrifuge tube designated for the 10X Genomics single-cell gene expression assay. Nuclei were centrifuged at 300 RCF for 5 min at 4 °C, and supernatant was aspirated. For ATAC, nuclei were resuspended in Diluted Nuclei Buffer (10X Genomics). For the gene expression assay, nuclei were resuspended in Wash and Resuspension Buffer (1X PBS, 1% BSA, 0.2 U/μL RNase Inhibitor). Nuclei were then filtered through a 35 μm strainer. Nuclei concentrations determined using a Countess™ II Automated Cell Counter (ThermoFisher) and nuclei quality was assessed at 10X and 40X magnification using an Evos XL Core Cell Imager (ThermoFisher).

### Parallel snATAC-seq/snRNA-seq library preparation and sequencing

snATAC-seq libraries were constructed as previously [57] using the Chromium Next GEM Single Cell ATAC Library and Gel Bead v1.1 kit, Chip H Single Cell kit, and Single Index Kit N Set A (10X Genomics) according to manufacturer’s instructions. In parallel, from the same pool of nuclei from each sample, snRNA-seq libraries were constructed using the Chromium Next GEM Single Cell 3’ GEM, Library, and Gel Bead v3.1 kit, Chip G Single Cell kit, and i7 Multiplex kit (10X Genomics) according to manufacturer’s instructions. For each sample, 10,000 nuclei were targeted for both the ATAC and 3′ assays. Library quality control was performed on a Bioanalyzer (Agilent) with the High Sensitivity DNA Kit (Agilent) according to manufacturer’s instructions and the 10X Genomics protocols. Libraries were submitted to the Sequencing and Genomic Technologies Shared Resource at Duke University for quantification using the KAPA Library Quantification Kit for Illumina® Platforms and sequencing. Groups of four snRNA-seq libraries were pooled on a NovaSeq 6000 S1 50 bp PE full flow cell to target a sequencing depth of 400 million reads per sample (Read 1 = 28, i7 index = 8, and Read 2 = 91 cycles). Groups of four snATAC-seq libraries were pooled on a NovaSeq 6000 S1 100 bp PE full flow cell to target a sequencing depth of 400 million reads per sample (Read 1N = 50, i7 index = 8, i5 index = 16, and Read 2N = 50 cycles). Sequencing was performed blinded to age, sex, and diagnosis.

### snRNA-seq data processing

Raw snRNA-seq sequencing data were converted to FastQ format, aligned to a GRCh38 pre-mRNA reference, filtered, and counted using CellRanger 4.0.0 (10X Genomics). Subsequent processing was done using Seurat 4.0.1 [92]. Filtered feature-barcode matrices were used to generate Seurat objects for the 24 samples. For QC filtering, nuclei below the 1st and above the 99th percentile for number of features were excluded. Nuclei above the 95th percentile for mitochondrial gene transcript proportion (or > 5% mitochondrial transcripts if 95th percentile mitochondrial transcript proportion was < 5%) were also excluded. Because experiments were conducted in nuclei rather than whole cells, mitochondrial genes were subsequently removed. The 24 Seurat objects were merged into one, and were iteratively normalized using SCTransform [93] with glmGamPoi, which alleviates bias from weakly-expressed genes [94]. Batch correction was performed using reference-based integration [22] on the 24 normalized datasets, which improves computational efficiency for integration.

### Cell type and subtype cluster annotation

Cell type annotation was conducted using a label transfer method [22] and a previously annotated reference dataset from human M1. Batch-corrected data from both our dataset and the human M1 dataset were used for label transfer. Nuclei with maximum prediction scores of < 0.5 were excluded. Nuclei with a percent difference of < 20% between first and second highest cell type prediction scores were termed “hybrid” and excluded [95]. Endothelial cells and VLMCs were in low abundance and did not form distinct UMAP clusters and were thus excluded. Following PCA, dimensionality was examined using an Elbow plot and by calculating variance contribution of each PC. UMAP was then run using the first 30 PCs, and nuclei were clustered based on UMAP reduction at a resolution of 0.7. This resolution level for cluster delineation was selected after comparison of a range of values as it was determined to provide optimal distinction between populations of nuclei displaying unique gene expression profiles as evidenced by their separation from one another in UMAP space. Counts of predicted major cell types based on the label transfer were examined for each of the 36 clusters (Table S2), and clusters were manually annotated based on the majority cell type for each cluster (e.g., ‘Exc1’, ‘Exc2’, etc.). Most frequent cell types comprised over 90% of cells in each cluster with the exceptions of Astro4, Micro2, Micro3 and OPC2 (Table S2).

### Human M1 reference data processing

To optimize label transfer, we re-processed previously published human primary motor cortex (M1) snRNA-seq data [23] to map it to GRCh38 Ensembl 80 as we did with our data [57]. FastQ files were obtained from the Neuroscience Multi-omic Data Archive (NeMO: https://nemoarchive.org/) and were aligned to the same GRCh38 pre-mRNA reference used for our data, filtered, and counted using CellRanger 4.0.0 (10X Genomics). Filtered feature-barcode matrices were used to generate separate Seurat objects for each sample, with nuclei absent from the annotated metadata excluded. Seurat objects were merged and iteratively normalized using SCTransform [93] with glmGamPoi. Batch correction was performed using reference-based integration [22] on the normalized datasets. The 127 transcriptomic cell types in this data were grouped into 8 broad cell types including astrocytes, endothelial cells, excitatory neurons, inhibitory neurons, microglia, oligodendrocytes, OPCs, and VLMCs.

### Cell type proportion comparisons

For both snRNA-seq and snATAC-seq datasets, proportion comparisons of cell types and subtypes between PD and Normal groups were made using the MASC algorithm [28]. Proportions were calculated by counting nuclei of each cell type and subtype and dividing this number by the total number of nuclei for each sample. For snRNA-seq data, age, sex, PMI, number of nuclei after QC filtering, median genes per cell, and average library size were included as fixed effects for MASC and sample donor ID was included as a random effect. For snATAC-seq data, age, sex, PMI, and number of nuclei after QC filtering were included as fixed effects for MASC and sample donor ID was included as a random effect. Benjamini–Hochberg correction for multiple testing was applied to MASC-derived *p* values.

### snATAC-seq data processing

As described previously [57], DNA fragments acquired from snATAC-seq were sequenced and converted to FastQ format, and then mapped to GENCODE’s human release 32 reference [96] and counted using CellRanger-ATAC 1.2.0 (10X Genomics). Remaining nuclei were screened using the following QC metrics: (I) Nucleosome signal: Ratio of mononucleosome fragments (147 to 294 bp) to nucleosome free fragments (< 147 bp). Nuclei having a nucleosome signal of > 4 were excluded [97]; (II) Transcription start site (TSS) enrichment: Ratio of aggregated, normalized read signal centered around a reference set of TSS’s compared to signal in the TSS flanking regions. Nuclei with TSS enrichment scores < 2 were excluded [97]; (III) Percent reads in peaks: Proportion of fragments per cell mapping to peak regions. Cells with < 15% of reads in peaks were excluded [97]; (IV) Total peak region fragments: Cells with < 1000 peak region fragments were excluded due to low sequencing depth. Additionally, cells in the upper 1% in each sample distribution were excluded as a precaution against multiplets [97]; (V) Blacklist ratio: Proportion of fragments mapping to sequences associated with technical artifacts. Cells with > 5% of fragments mapping to blacklisted regions were excluded [98].

Seurat 4.0.1 [22], Signac 1.3.0 [97], and Harmony 0.1.0 [99] R packages were used for subsequent processing. Latent semantic indexing (LSI) was used to create a low-rank approximation of the data [100]. The 24 datasets were term frequency-inverse document frequency (TF-IDF) normalized and aggregated to form a joint peak-by-cell count matrix. Singular value decomposition (SVD) was then performed on this dataset, followed by standardization of left singular vectors, representing LSI components. Correlation of each component with sequencing depth was then measured. The first dimension was removed from downstream analysis due to high sequencing depth correlation (rho = 0.7) [97]. Remaining LSI components were then adjusted with using the *RunHarmony* function to remove batch effects prior to data clustering. As in snRNA dimensionality reduction, we used dimensions 2 through 30 for clustering and subsequent analysis.

Clusters were constructed from the adjusted LSI embeddings of the integrated dataset using the *FindNeighbors* and *FindClusters* Seurat functions, with *k*-nearest neighbors = 20, and cluster resolution = 1. The data were then projected onto a 2D surface via UMAP to evaluate cluster resolution.

### Cell type annotation of snATAC-seq nuclei

As described previously [57], cell type annotation of the snATAC cells was performed using the integrated snRNA dataset as a reference [22]. “Gene activity” matrices were constructed for each nucleus from snATAC samples by counting fragments mapped to gene promoters (2000 bp upstream to 200 bp downstream of TSS). These matrices were log-normalized following promoter region fragment quantifications. The *FindTransferAnchors* Seurat function was then employed to annotate the snATAC data using the snRNA data as reference, with reduction via canonical correlation analysis. This entails computation of cross correlation between snATAC and snRNA cell variable features. After L2 normalization, the left and right singular vectors from the SVD of this matrix are taken as the canonical correlation vectors. A mutual nearest neighbor approach is then used to find anchors between the datasets, representing biologically similar cell states across modalities. Major cell type prediction scores were calculated for each nucleus using the weighted combination of the *k*-nearest anchors. The cell type with the maximum prediction score was the predicted cell type identity. Nuclei with maximum prediction scores of < 0.5 were excluded. “Hybrid” nuclei were identified using the same metric as for snRNA-seq data above, and excluded.

### Doublet/multiplet detection in snRNA-seq and snATAC-seq data

Multiplets comprising different cell types (heterotypic) were excluded from snRNA and snATAC data by considering the “hybrid score”, as described previously [57]. The hybrid score is calculated as (x_1_ − x_2_)/x_1_, where x_1_ is the highest and x_2_ is the second highest prediction score [95]. Heterotypic multiplets would be expected to exhibit competing cell type prediction scores due to the presence of transcriptomic/epigenomic profiles from multiple cell types. Multiplets composed of one cell type (homotypic) were identified based on the number of features per cell (snRNA) or the total number of fragments in peaks (snATAC). snRNA nuclei with feature counts > 99th percentile and snATAC nuclei with total fragments in peaks > 95th percentile were excluded. Methods for Removal of homotypic multiplets in this manner is expected to also aid in filtering of heterotypic multiplets.

### Linking snATAC and snRNA datasets

The *FindTransferAnchors* and *TransferData* Seurat functions were used to link snATAC clusters to snRNA clusters, as described previously [57]. Anchors were used to transfer snRNA cluster information to snATAC nuclei as in cell type annotation. Each snATAC nucleus was given 36 prediction scores, corresponding to each snRNA cluster. The snRNA cluster prediction scores were summed across all nuclei within each snATAC cluster. Each snATAC cluster was assigned a closest matching snRNA cluster based on its maximum prediction score. To ensure concordance, the cell types of the linked snRNA clusters were compared to the original snATAC cluster cell types. To assess the accuracy of the cluster linking methodology, we previously used PBMC granulocyte multiome data, freely available on the 10X Genomics website, estimating the average accuracy of such cluster linking to be approximately 75.6%, and found that this level of certainty was sufficient to provide meaningful insight into the relationships between epigenetic and transcriptomic observations [57].

### Peak calling

As previously [57], peak regions were predicted empirically within each sample separately using the MACS2 algorithm on each cluster [101]. Peaks were designated as regions having a *p* value of ≤ 10e-5. The Multi Sample Peak Calling (MSPC) software package was used [102] to combine peaks into a consensus set for each cluster, with Fisher’s method used to evaluate overlapping peaks across samples. Peaks occurring in at least 2 samples, with an FDR of ≤ 0.05 from Fisher’s combined probability test were used as the consensus set for downstream analysis.

### Covariate selection for differential analyses

Prior to differential analysis, as previously [57] we estimated the impact of multiple technical variables as well as donor-level characteristics separately for the snRNA-seq (Table S1) and snATAC-seq (Table S5) experiments. Read counts were summed for all nuclei in each donor sample, resulting in only one expression/accessibility value per sample per gene/peak, as all nuclei from a particular donor would have identical donor characteristics. Genes with no expression or peaks with zero accessibility for > 20% of samples were subsequently removed, and all values were mean-centered and scaled prior to covariate analysis. PCA was then performed for genes and peaks using *prcomp* in R. We then carried out linear regression using *glm* in R for PCs explaining > 10% of the variability in global expression or chromatin accessibility on both nuclei- and donor-specific metadata variables to identify factors that should be included as covariates in differential analyses. Specifically, we selected the variable most associated (surpassing Bonferroni correction for multiple testing, *q* < 0.05) with PC1 (or alternatively, the PC explaining the most variability) and regressed all genes or peaks on the associated variable to obtain gene or peak residuals that are adjusted for its effect. We then performed PC analysis on the gene or peak residuals, and in an iterative process, repeating the above steps until no additional metadata variables were associated with global expression or chromatin accessibility (*q* < 0.05). For snRNA-seq data, age, sex, PMI, number of nuclei after QC filtering, median genes per cell, and average library size were selected as covariates for differential expression gene analysis. For snATAC-seq data, age, sex, PMI, and number of nuclei after QC filtering were selected as covariates for differential peak accessibility analysis.

### Differential expression analysis

In order to identify DEGs at both the cell type and subtype levels between PD and Normal samples within our snRNA-seq dataset, we employed the NEBULA algorithm [29]. Specifically, the NEBULA-HL procedure was used as this process is optimized for estimating both nucleus-level and donor-level data overdispersions [29, 103]. Prior to running NEBULA, for each cell type and cluster, genes expressed in less than 10% of cells in either group (PD or Normal) were filtered out. For snRNA-seq data, age, sex, PMI, number of nuclei after QC filtering, median genes per cell, and average library size were included as fixed effects for NEBULA and sample donor ID was included as a random effect. The reference level was set to ‘Normal’ such that the results for log_2_FC coefficients would be positive if up-regulated in PD and negative if down-regulated in PD. Benjamini–Hochberg correction for multiple testing was applied at the gene level to NEBULA-derived *p* values.

### Differential accessibility analysis

In order to identify DAPs at both the cell type and subtype levels between PD and Normal samples within our snATAC-seq dataset, The NEBULA-HL algorithm was used as above for DEG identification. Prior to running NEBULA, for each cluster, peaks accessible in less than 10% of cells in either group (PD or Normal) were filtered out. For snATAC-seq data, age, sex, PMI, and number of nuclei after QC filtering were included as fixed effects for NEBULA and sample donor ID was included as a random effect. The reference level was set to ‘Normal’ such that the results for log_2_FC coefficients would be positive if more accessible in PD and negative if less accessible in PD. Benjamini–Hochberg correction for multiple testing was applied at the peak level to NEBULA-derived *p* values.

### Cluster marker identification

To characterize the transcriptional profile of Exc5 cells, we used the FindMarkers function of the Seurat R package, employing a Wilcoxon Rank Sum test, to compare gene expression in Exc5 nuclei to nuclei of all other excitatory neuron clusters, thereby identifying marker genes defining this cluster. In order to focus on the most significant marker genes, we used a log_2_FC threshold of > 0.25 and a gene-level FDR-adjusted *p*-value threshold of < 0.01, and identified 432 positive (upregulated) marker genes, and 498 negative (downregulated) marker genes for the Exc5 cluster (Table S4).

### Biological pathway enrichment analysis

In order to understand the biological significance of gene sets derived from differential expression analyses, we employed the Metascape [30] algorithm (metascape.org). The gene set of interest was input as the target gene list, and the total set of genes examined in the corresponding differential expression analysis was input as the background gene list. In order to group the top 20 enriched Metascape output GO terms into broader biological categories, functions of each individual gene (based on literature search) contributing to a particular category were qualitatively grouped into major functional categories. If a majority of the target genes associated with an enriched pathway GO term were associated with a particular functional category, this category was assigned to the GO term. A single GO term could be assigned to multiple functional categories. For analyses of shared pathways among clusters, Micro3 was excluded, as the majority cell type in this cluster represented below 50 percent of total nuclei in the cluster (Table S2).

### Assessment of cCREs and CCANs

We used the R package Cicero [56] to characterize chromatin interactions in the data using as done previously [57]. The Cicero pipeline was conducted on a per cluster basis using PD cells only. Integrated LSI embeddings were passed to Cicero’s bootstrap aggregation process, wherein highly similar cells are aggregated by summing the raw counts in groups of 50k-nearest neighbors. Fragment sums are then normalized to account for within-group sequencing depth. A graphical LASSO is then used to estimate the partial correlation structure of each peak with neighboring peaks. A penalty term based on the genomic distance between peak pairs is used in GLASSO, and the resulting regularized correlations derived from the precision matrix are termed “coaccessibility scores.” The maximum peak-peak distance, at which regularized correlations are assigned 0, was defined as 500 Kb. A minimum coaccessibility score of 0.2 was specified before extracting CCANs from the resulting data via Louvain community detection. A coaccessibility score of ≥ 0.2 was previously shown to provide a reliable indication of meaningful peak pair accessibility correlation, and was thus used as our threshold for defining coaccessible peaks [56, 57]. Each peak within a CCAN was coaccessible with at least one other peak in the network. cCREs were defined as peaks within 500 kb of and with minimum coaccessibility scores of 0.2 with peaks overlapping the promoter (2 kb region upstream of start codon) or intron 1 regions of target genes.

### Motif detection and enrichment analysis

To detect TFBS in the data, the genome was scanned for motifs within cCREs (including distal cCRE peaks and promoter/intron1 peaks) of DEGs of interest, using position weight matrices (PWMs) from JASPAR 2020 [104, 105]. A *p* value threshold of 5e-5 was used for motif matches. In the case of overlapping motif matches of the same TF, only the highest scoring match was used. We then used HOMER to detect motif enrichment in cCREs [61]. HOMER first quantifies the GC content and *n*-mer composition of both the background and target regions and applies weights to eliminate sequence bias before using a binomial test to compute enrichment *p*-values. Peaks within cCREs were used as target sequences, whereas all other cluster-specific peaks were used as background regions. For downstream analysis, we used only enriched motifs with FDR ≤ 0.05 and fold enrichment ≥ 1.2.

### Definition of loci and SNVs for analysis

Loci for analysis of SNV impact on TF binding sites were defined by the cell-specific clusters for each cell type (microglia, oligodendrocyte precursor cells, inhibitory neurons) that were determined from the PD-GWAS data [5]. Identity of the cell-type and associated GWAS gene were retained for all subsequent analysis steps. SNVs located within the cCREs were catalogued from dbSNV version 151 with a global MAF ≥ 0.01. Genomic location, reference and alternate alleles were obtained from dbSNV and used for the TF analysis.

### In silico prediction of TF binding sites affected by genetic variants in PD

Prediction of TF binding sites was completed for 1175 sequence variants, including, 1035 SNVs, 27 insertions, and 113 deletions. The software package/algorithm *motifbreakR* [63] (version 2.15) was used to estimate or predict whether the sequence surrounding a variant matches to specific TF binding sites, and how one allele of the variant relative to the other affects the strength of the TF binding site (gain or loss of the TF binding affinity). *MotifbreakR* can predict effects for novel or previously described variants in public databases. TF binding affinity scores were compared for all variants with at least 1% frequency within the database population using the 1000 Genomes database of human genetic variation [64]. For this study, we utilized the information content (ic) algorithm and position weight matrices from Homer, HOCOMOCO, Factorbook and ENCODE. Each variant from the catalogue we generated from the cell-specific PD-GWAS was evaluated for the potential to disrupt/gain TF binding sites using a predicted *p* value < 1 × 10^–4^. The variants were evaluated for impact on specific TF binding with calculation of a permutation *p* value, score for impact on binding and assessment of loss or gain of a binding site based on the *motifbreakR* calculations.

### Linkage disequilibrium (LD) calculations

LD statistics (r^2^ and D′) between the PD GWAS-SNV and the specific variant that disrupts the transcription factor binding site using the LD matrix tool option from the LD Link software [106]. European panels were used to correspond to the ancestries covered in the PD GWAS. JMP software (SAS Institute, Cary, NC, version 17.0.0) was used to complete the annotation of the pairwise LD results for all of the PD GWAS SNVs and corresponding SNVs in the transcription factor binding sites.

### Genome version and coordinates

All genomic data and coordinates are based on the December 2013 version of the genome: hg38, GRCh38.

### Supplementary Information

Supplementary material 1: Supplementary figures S1–S10 

Supplementary material 2. Supplementary tables S1–S13

## Data Availability

The snRNA-seq and snATAC-seq data are available at the Synapse data repository (https://synapse.org, ProjectSynID: syn60245188 and syn50996869). Access will be avaliable upon request under controlled use conditions. In addition, the snRNA-seq and snATAC-seq raw and normalized count data generated in this study are available at the Duke Research Data Repository (https://research.repository.duke.edu). All computer code used for this study has been deposited to GitHub (https://www.github.com) and will be made available upon request.
