# Supplementary material for: Single-nucleus multi-omics of Parkinson’s disease reveals a glutamatergic neuronal subtype susceptible to gene dysregulation via alteration of transcriptional networks
Source: Acta Neuropathol Commun. 2024 Jul 2;12:111. doi: 10.1186/s40478-024-01803-1 (PMC11218415; doi:10.1186/s40478-024-01803-1)
Supplement: Supplementary file 1 — Supplementary material 1: Supplementary figures S1–S10 [file 40478_2024_1803_MOESM1_ESM.pptx]

## Slide 1
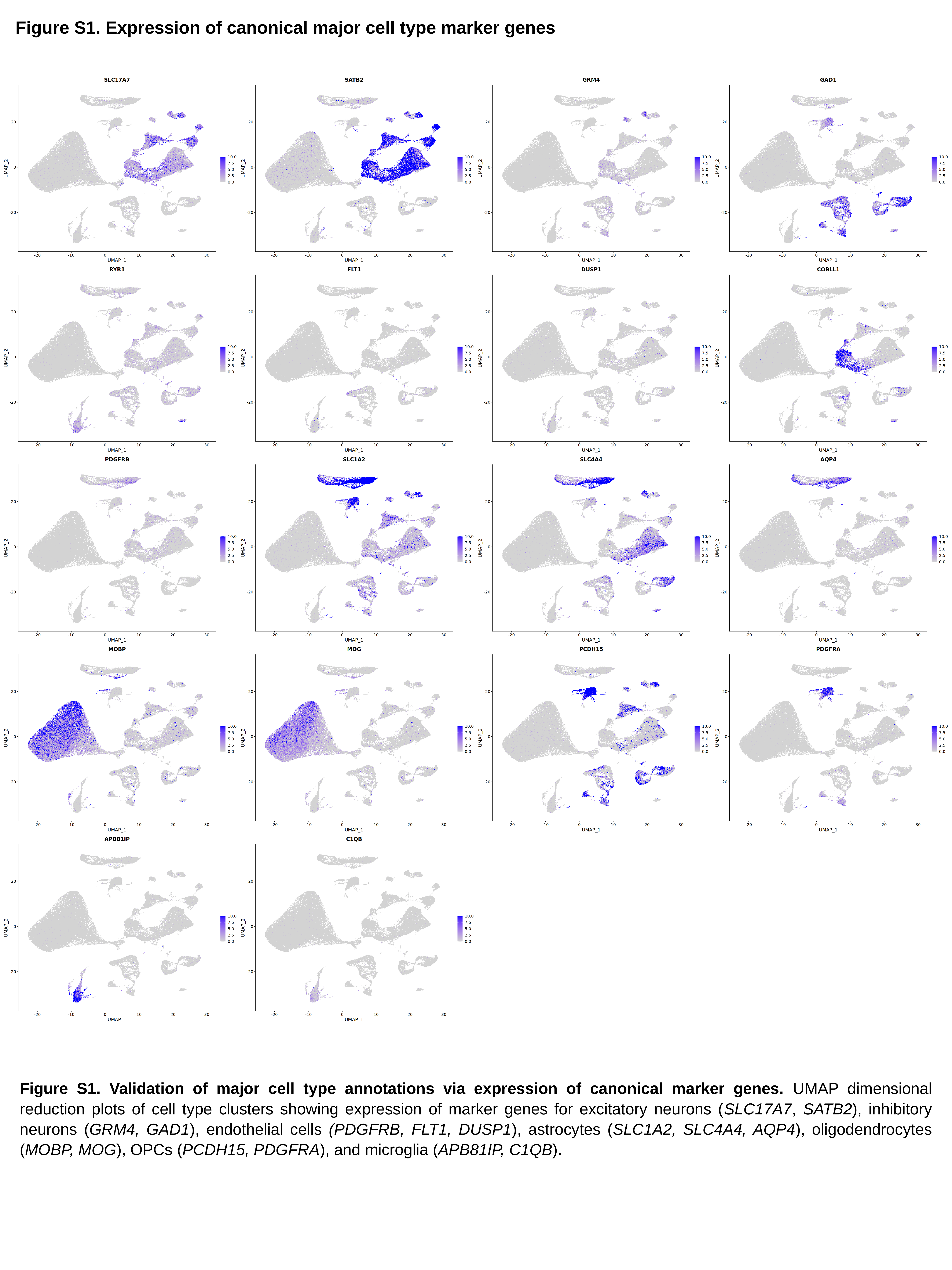

Figure S1. Expression of canonical major cell type marker genes
Figure S1. Validation of major cell type annotations via expression of canonical marker genes. UMAP dimensional reduction plots of cell type clusters showing expression of marker genes for excitatory neurons (SLC17A7, SATB2), inhibitory neurons (GRM4, GAD1), endothelial cells (PDGFRB, FLT1, DUSP1), astrocytes (SLC1A2, SLC4A4, AQP4), oligodendrocytes (MOBP, MOG), OPCs (PCDH15, PDGFRA), and microglia (APB81IP, C1QB).

## Slide 2
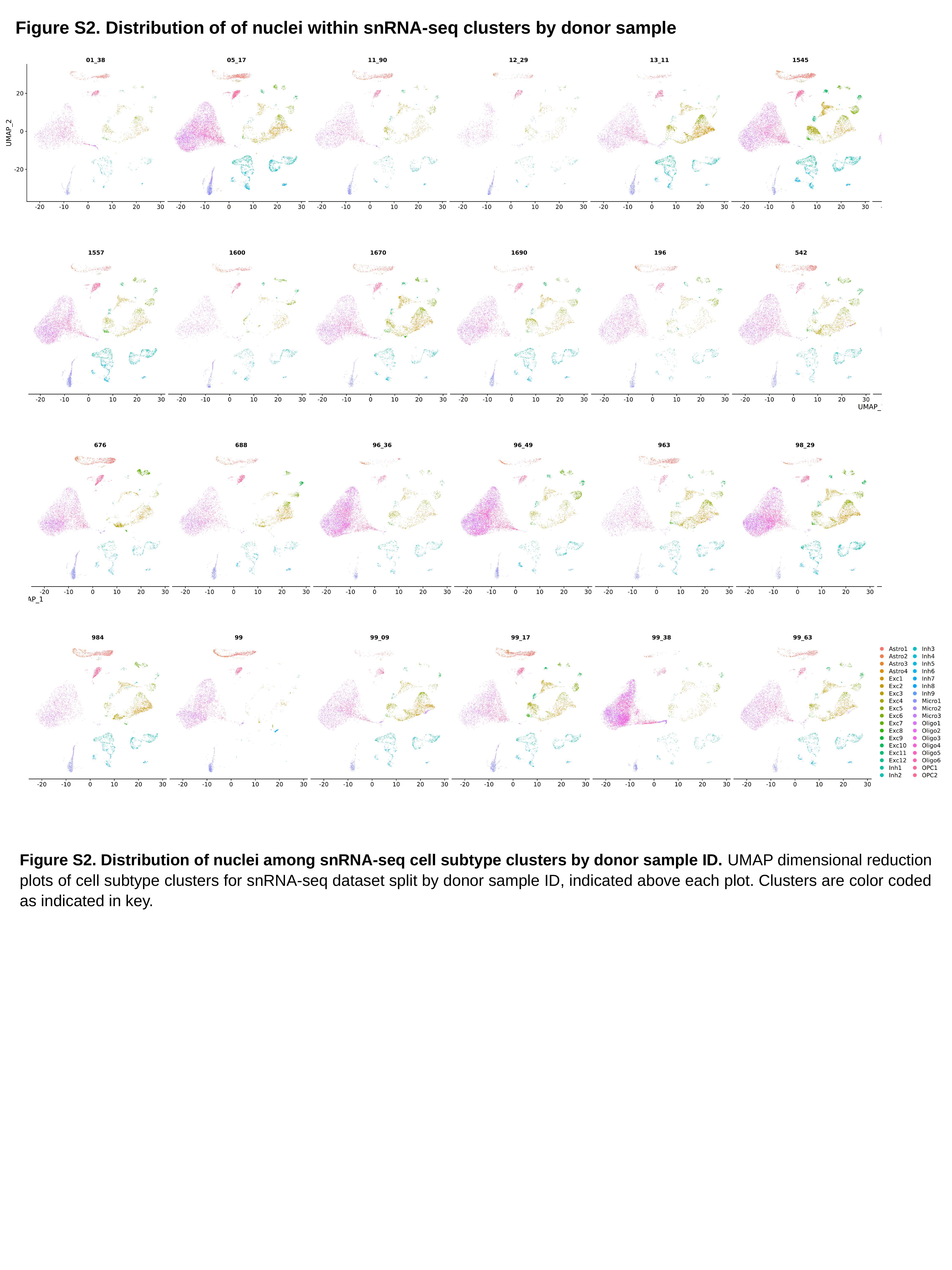

Figure S2. Distribution of of nuclei within snRNA-seq clusters by donor sample
Figure S2. Distribution of nuclei among snRNA-seq cell subtype clusters by donor sample ID. UMAP dimensional reduction plots of cell subtype clusters for snRNA-seq dataset split by donor sample ID, indicated above each plot. Clusters are color coded as indicated in key.

## Slide 3
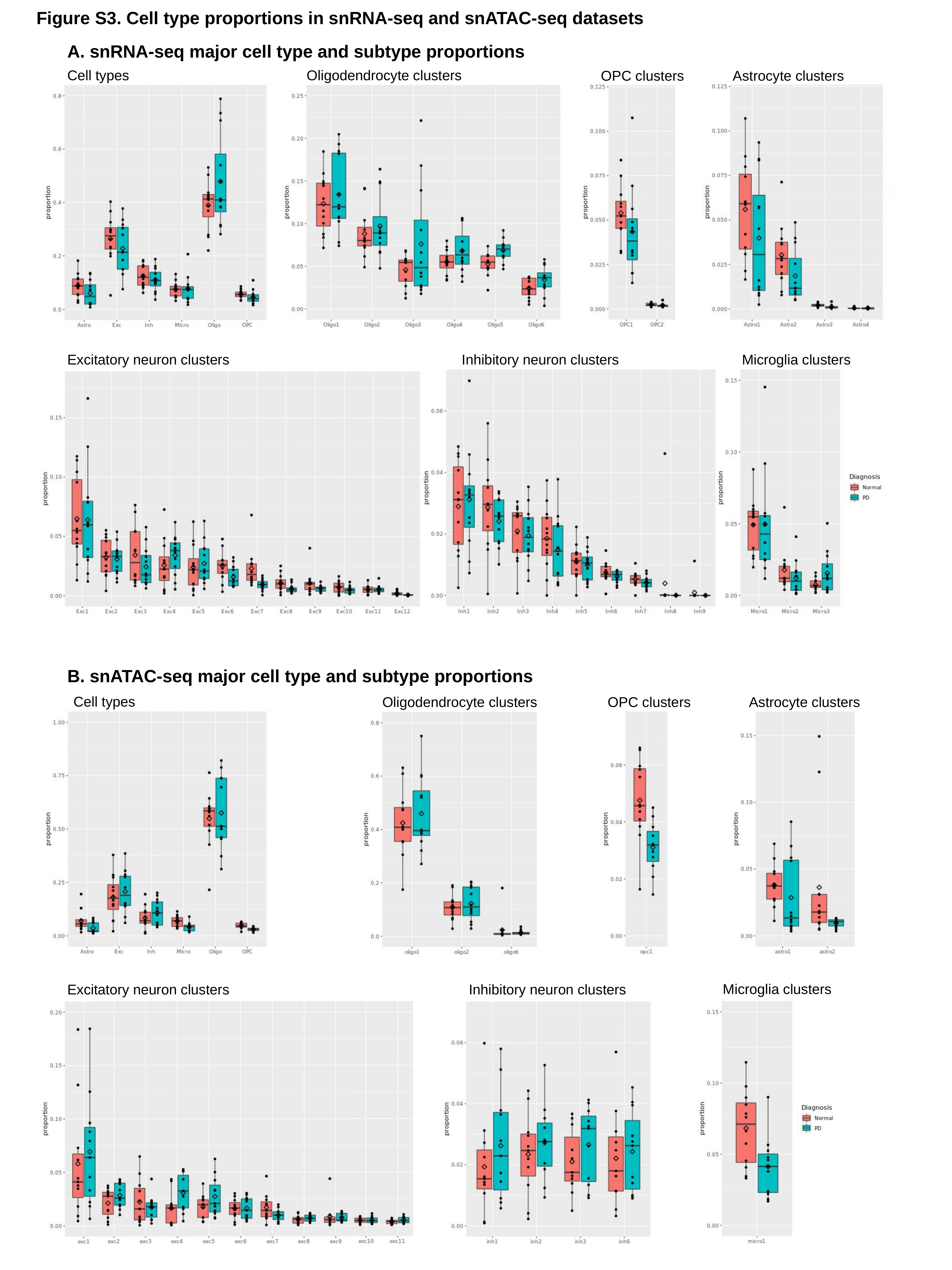

Figure S3. Cell type proportions in snRNA-seq and snATAC-seq datasets
A. snRNA-seq major cell type and subtype proportions
Cell types
Oligodendrocyte clusters
OPC clusters
Astrocyte clusters
Excitatory neuron clusters
Inhibitory neuron clusters
Microglia clusters
B. snATAC-seq major cell type and subtype proportions
Cell types
Oligodendrocyte clusters
OPC clusters
Astrocyte clusters
Microglia clusters
Excitatory neuron clusters
Inhibitory neuron clusters

## Slide 4
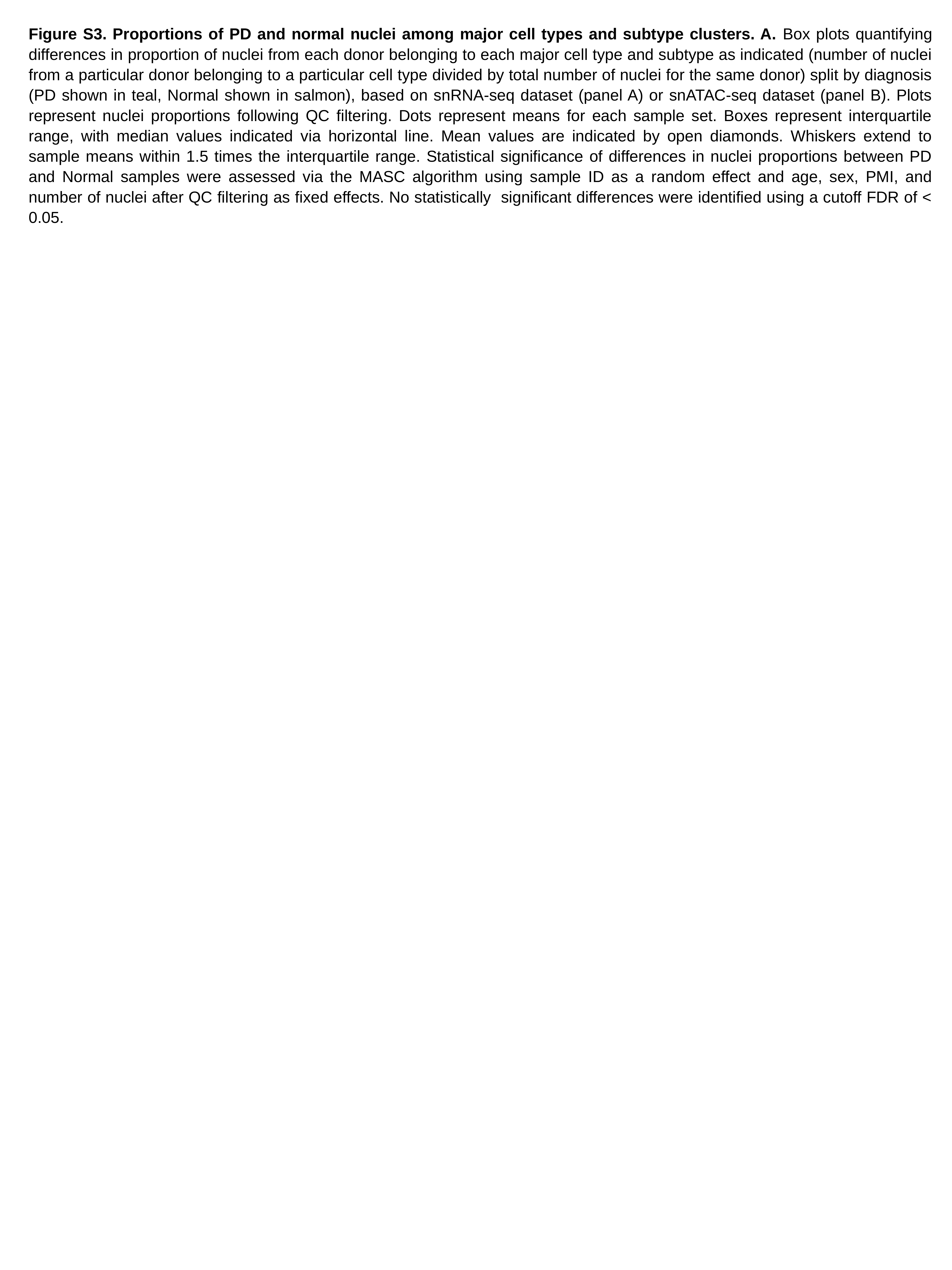

Figure S3. Proportions of PD and normal nuclei among major cell types and subtype clusters. A. Box plots quantifying differences in proportion of nuclei from each donor belonging to each major cell type and subtype as indicated (number of nuclei from a particular donor belonging to a particular cell type divided by total number of nuclei for the same donor) split by diagnosis (PD shown in teal, Normal shown in salmon), based on snRNA-seq dataset (panel A) or snATAC-seq dataset (panel B). Plots represent nuclei proportions following QC filtering. Dots represent means for each sample set. Boxes represent interquartile range, with median values indicated via horizontal line. Mean values are indicated by open diamonds. Whiskers extend to sample means within 1.5 times the interquartile range. Statistical significance of differences in nuclei proportions between PD and Normal samples were assessed via the MASC algorithm using sample ID as a random effect and age, sex, PMI, and number of nuclei after QC filtering as fixed effects. No statistically significant differences were identified using a cutoff FDR of < 0.05.

## Slide 5
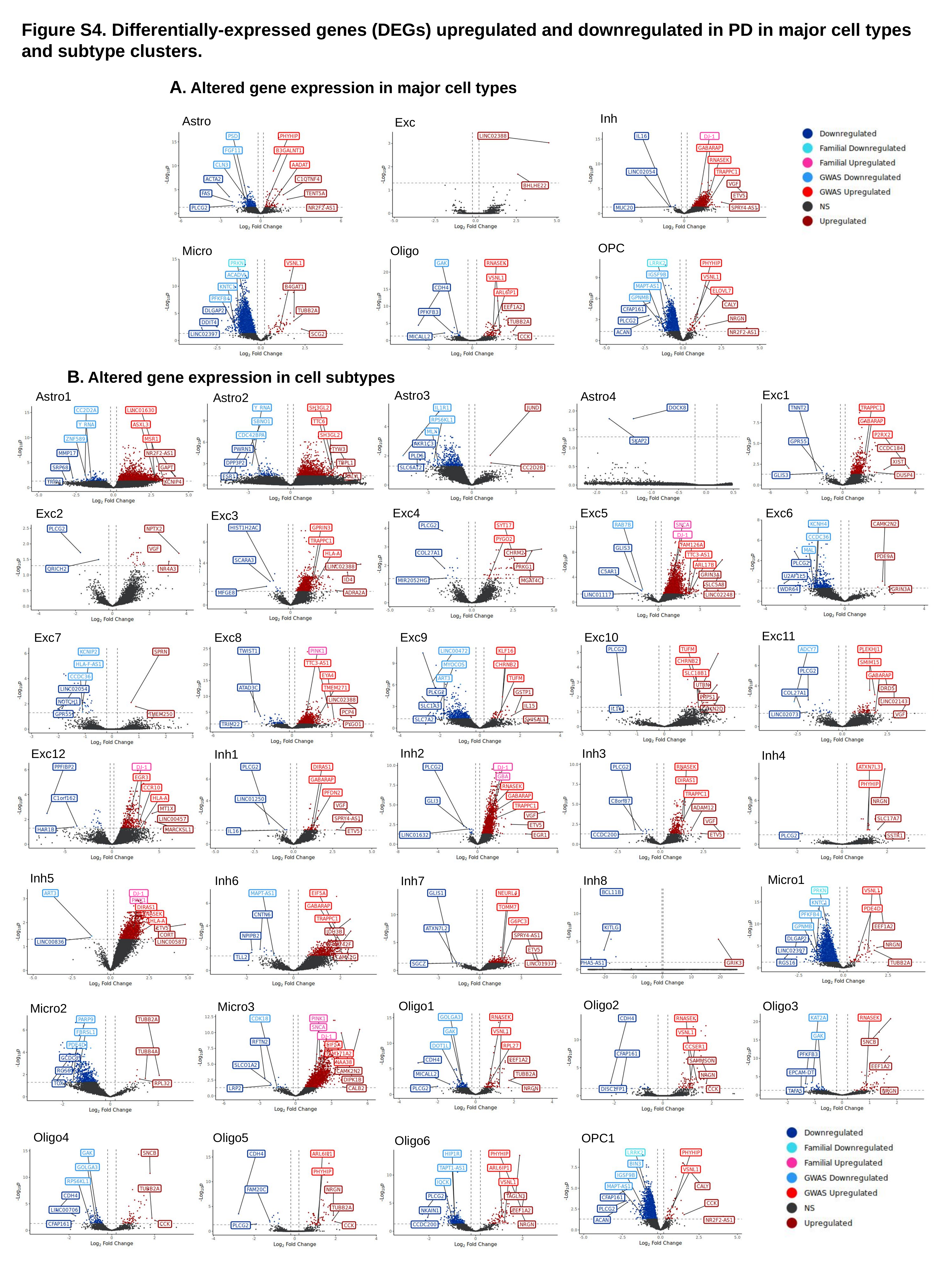

Figure S4. Differentially-expressed genes (DEGs) upregulated and downregulated in PD in major cell types and subtype clusters.
A. Altered gene expression in major cell types
Inh
Astro
Exc
DJ-1
OPC
Oligo
Micro
B. Altered gene expression in cell subtypes
Exc1
Astro3
Astro1
Astro4
Astro2
Exc6
Exc5
Exc4
Exc2
Exc3
DJ-1
Exc11
Exc9
Exc10
Exc8
Exc7
Inh2
Inh3
Exc12
Inh1
Inh4
DJ-1
DJ-1
Inh5
Micro1
Inh8
Inh6
Inh7
DJ-1
Oligo2
Oligo1
Oligo3
Micro3
Micro2
DJ-1
Oligo4
Oligo5
OPC1
Oligo6

## Slide 6
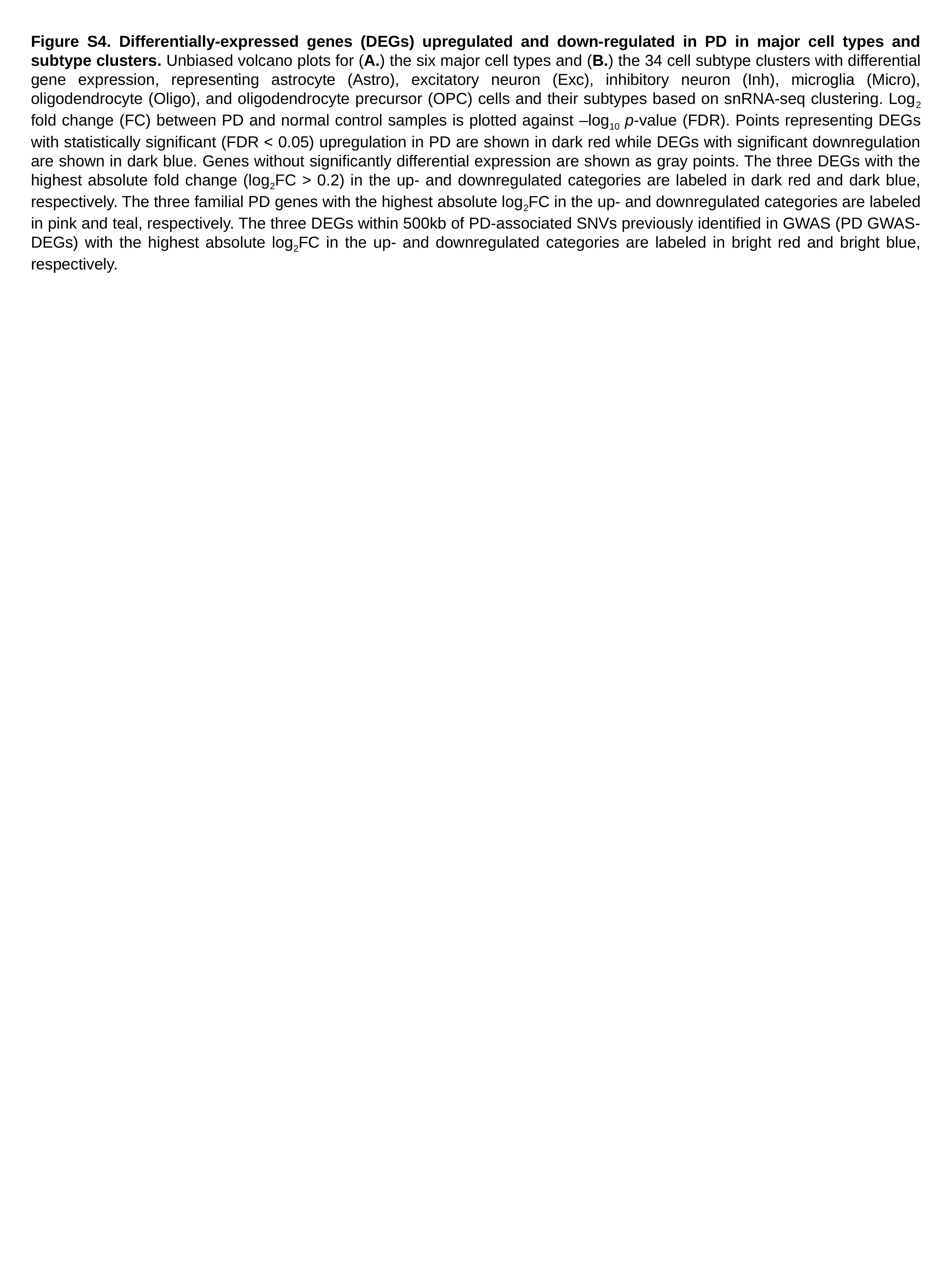

Figure S4. Differentially-expressed genes (DEGs) upregulated and down-regulated in PD in major cell types and subtype clusters. Unbiased volcano plots for (A.) the six major cell types and (B.) the 34 cell subtype clusters with differential gene expression, representing astrocyte (Astro), excitatory neuron (Exc), inhibitory neuron (Inh), microglia (Micro), oligodendrocyte (Oligo), and oligodendrocyte precursor (OPC) cells and their subtypes based on snRNA-seq clustering. Log2 fold change (FC) between PD and normal control samples is plotted against –log10 p-value (FDR). Points representing DEGs with statistically significant (FDR < 0.05) upregulation in PD are shown in dark red while DEGs with significant downregulation are shown in dark blue. Genes without significantly differential expression are shown as gray points. The three DEGs with the highest absolute fold change (log2FC > 0.2) in the up- and downregulated categories are labeled in dark red and dark blue, respectively. The three familial PD genes with the highest absolute log2FC in the up- and downregulated categories are labeled in pink and teal, respectively. The three DEGs within 500kb of PD-associated SNVs previously identified in GWAS (PD GWAS-DEGs) with the highest absolute log2FC in the up- and downregulated categories are labeled in bright red and bright blue, respectively.

## Slide 7
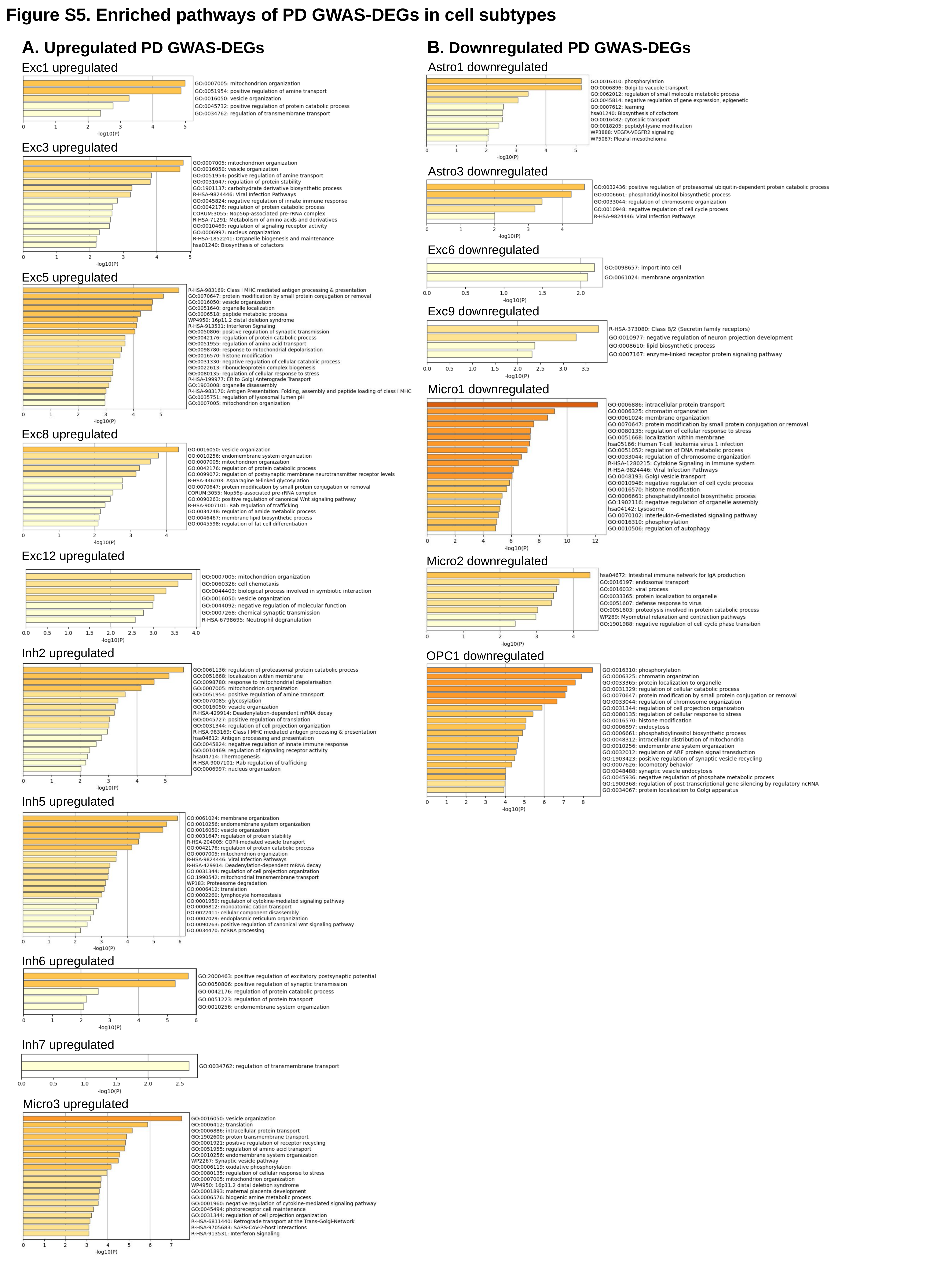

Figure S5. Enriched pathways of PD GWAS-DEGs in cell subtypes
A. Upregulated PD GWAS-DEGs
B. Downregulated PD GWAS-DEGs
Astro1 downregulated
Exc1 upregulated
Exc3 upregulated
Astro3 downregulated
Exc6 downregulated
Exc5 upregulated
Exc9 downregulated
Micro1 downregulated
Exc8 upregulated
Exc12 upregulated
Micro2 downregulated
Inh2 upregulated
OPC1 downregulated
Inh5 upregulated
Inh6 upregulated
Inh7 upregulated
Micro3 upregulated

## Slide 8
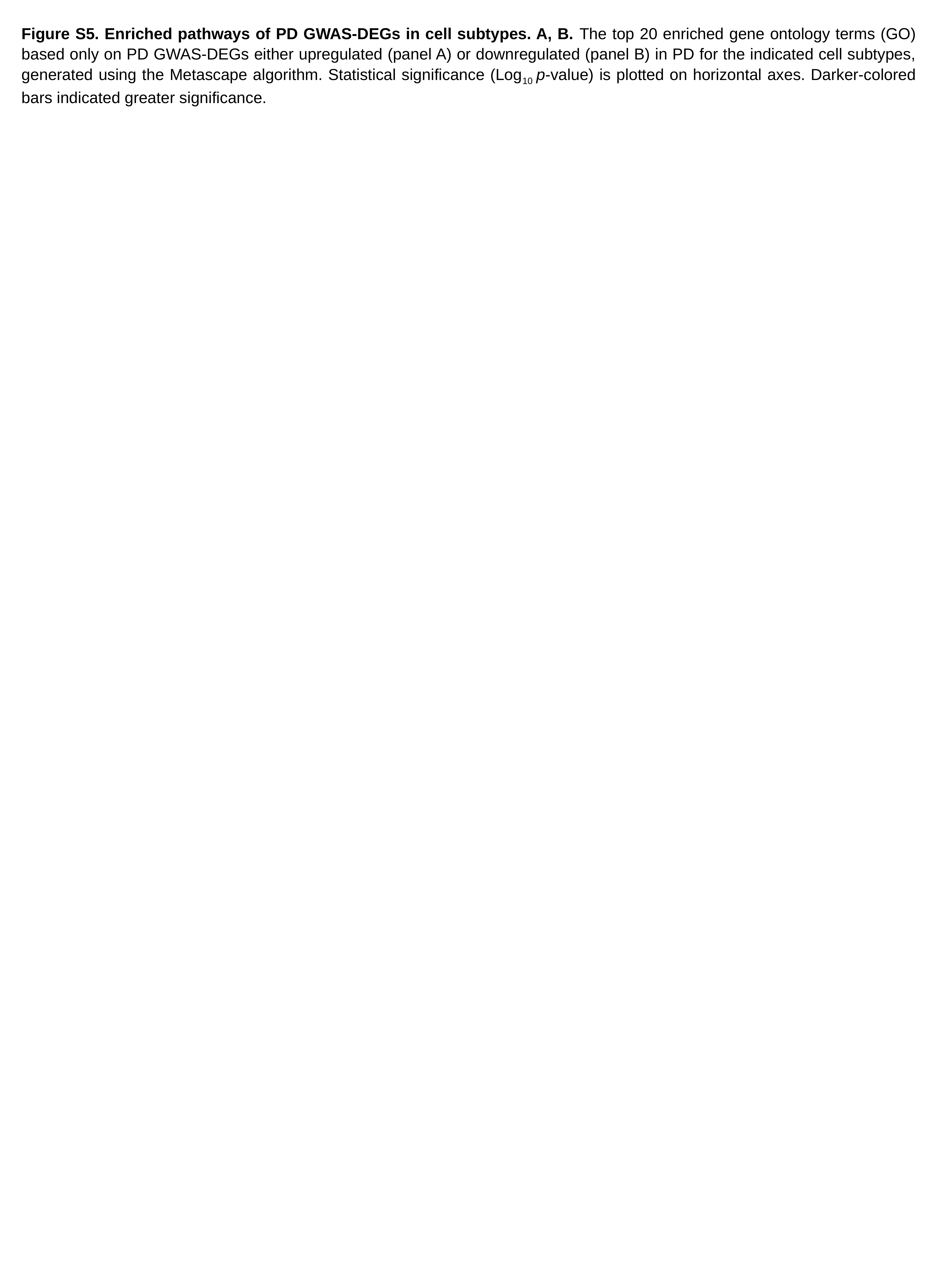

Figure S5. Enriched pathways of PD GWAS-DEGs in cell subtypes. A, B. The top 20 enriched gene ontology terms (GO) based only on PD GWAS-DEGs either upregulated (panel A) or downregulated (panel B) in PD for the indicated cell subtypes, generated using the Metascape algorithm. Statistical significance (Log10 p-value) is plotted on horizontal axes. Darker-colored bars indicated greater significance.

## Slide 9
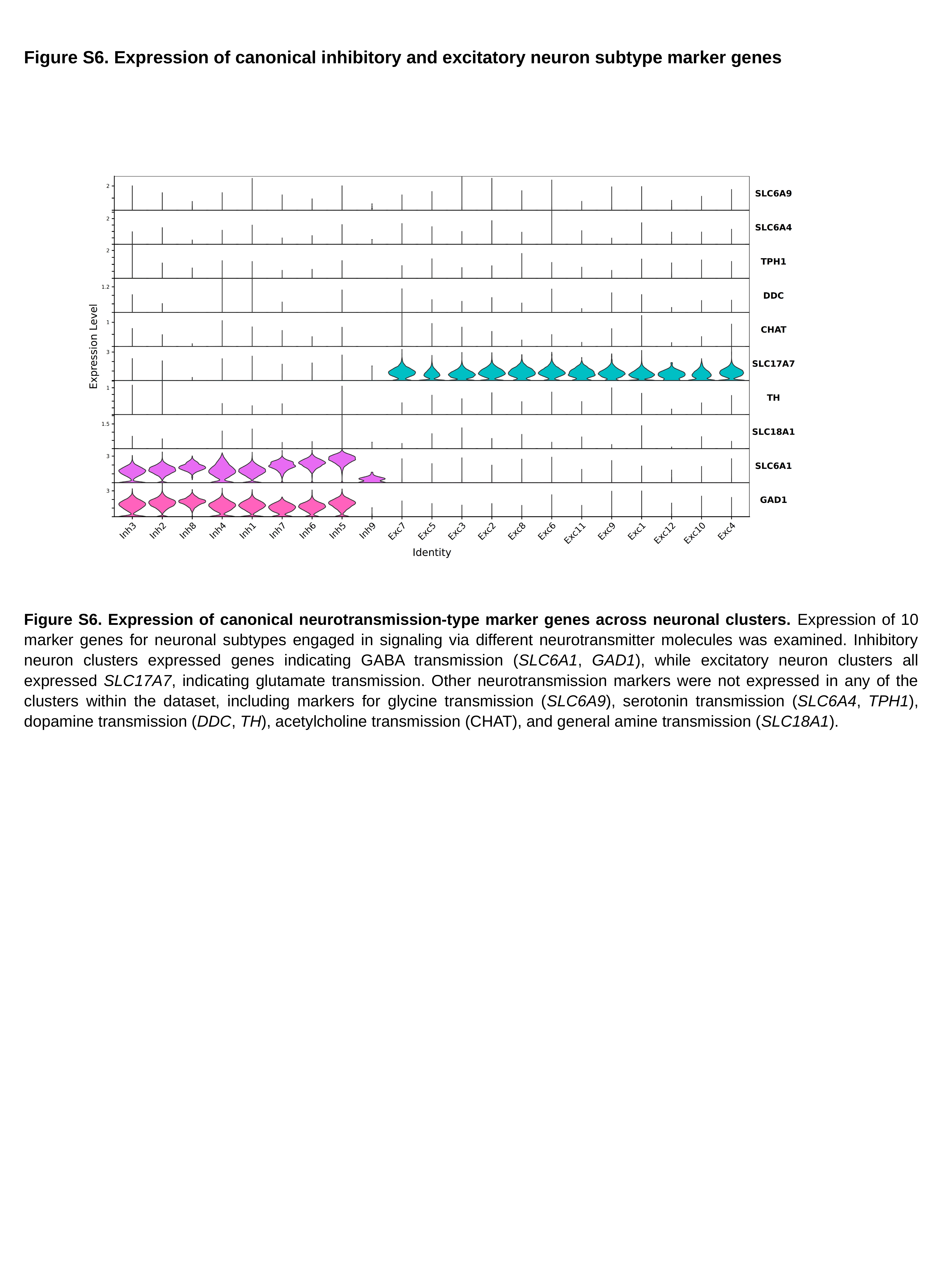

Figure S6. Expression of canonical inhibitory and excitatory neuron subtype marker genes
Figure S6. Expression of canonical neurotransmission-type marker genes across neuronal clusters. Expression of 10 marker genes for neuronal subtypes engaged in signaling via different neurotransmitter molecules was examined. Inhibitory neuron clusters expressed genes indicating GABA transmission (SLC6A1, GAD1), while excitatory neuron clusters all expressed SLC17A7, indicating glutamate transmission. Other neurotransmission markers were not expressed in any of the clusters within the dataset, including markers for glycine transmission (SLC6A9), serotonin transmission (SLC6A4, TPH1), dopamine transmission (DDC, TH), acetylcholine transmission (CHAT), and general amine transmission (SLC18A1).

## Slide 10
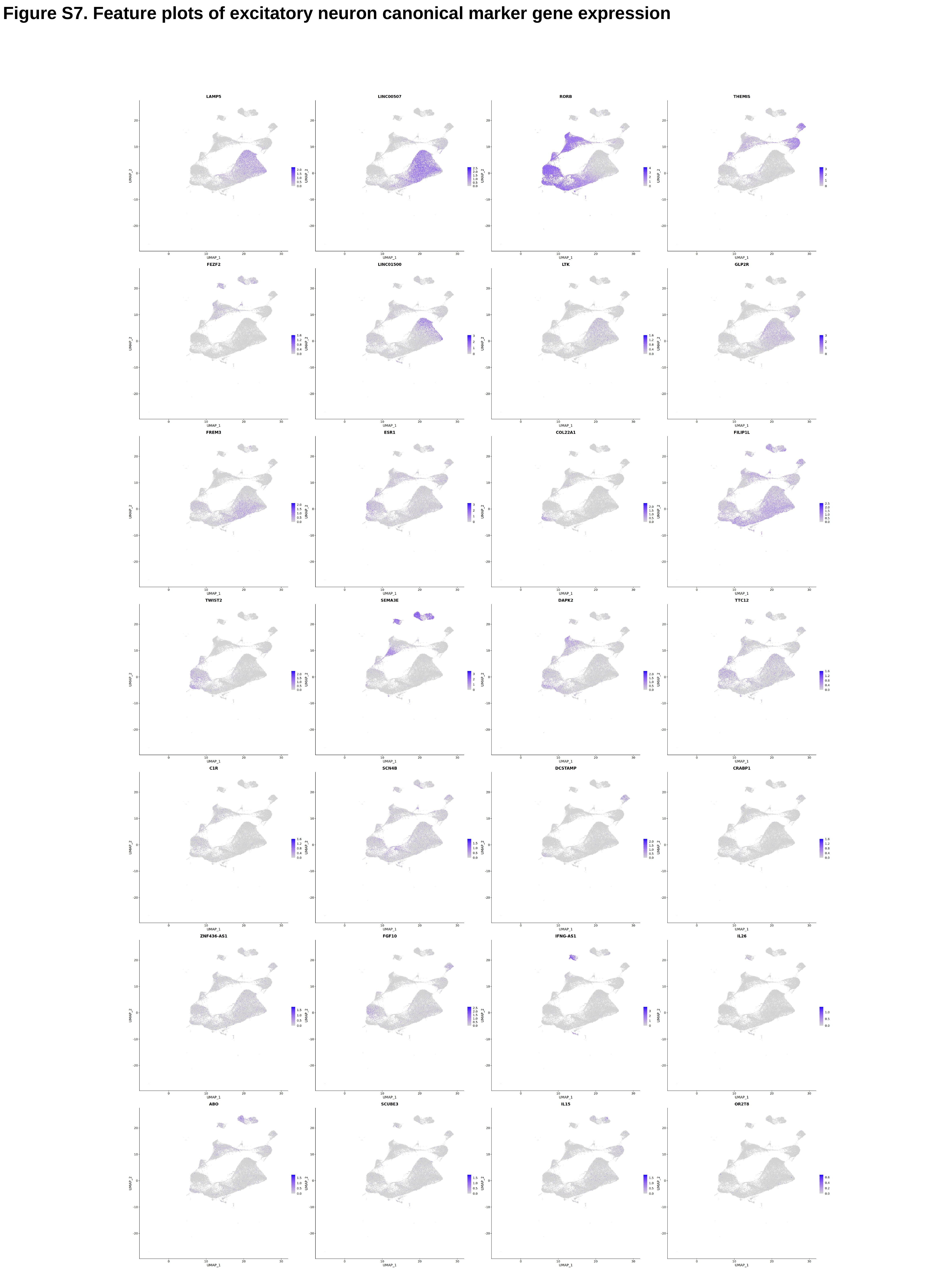

Figure S7. Feature plots of excitatory neuron canonical marker gene expression

## Slide 11
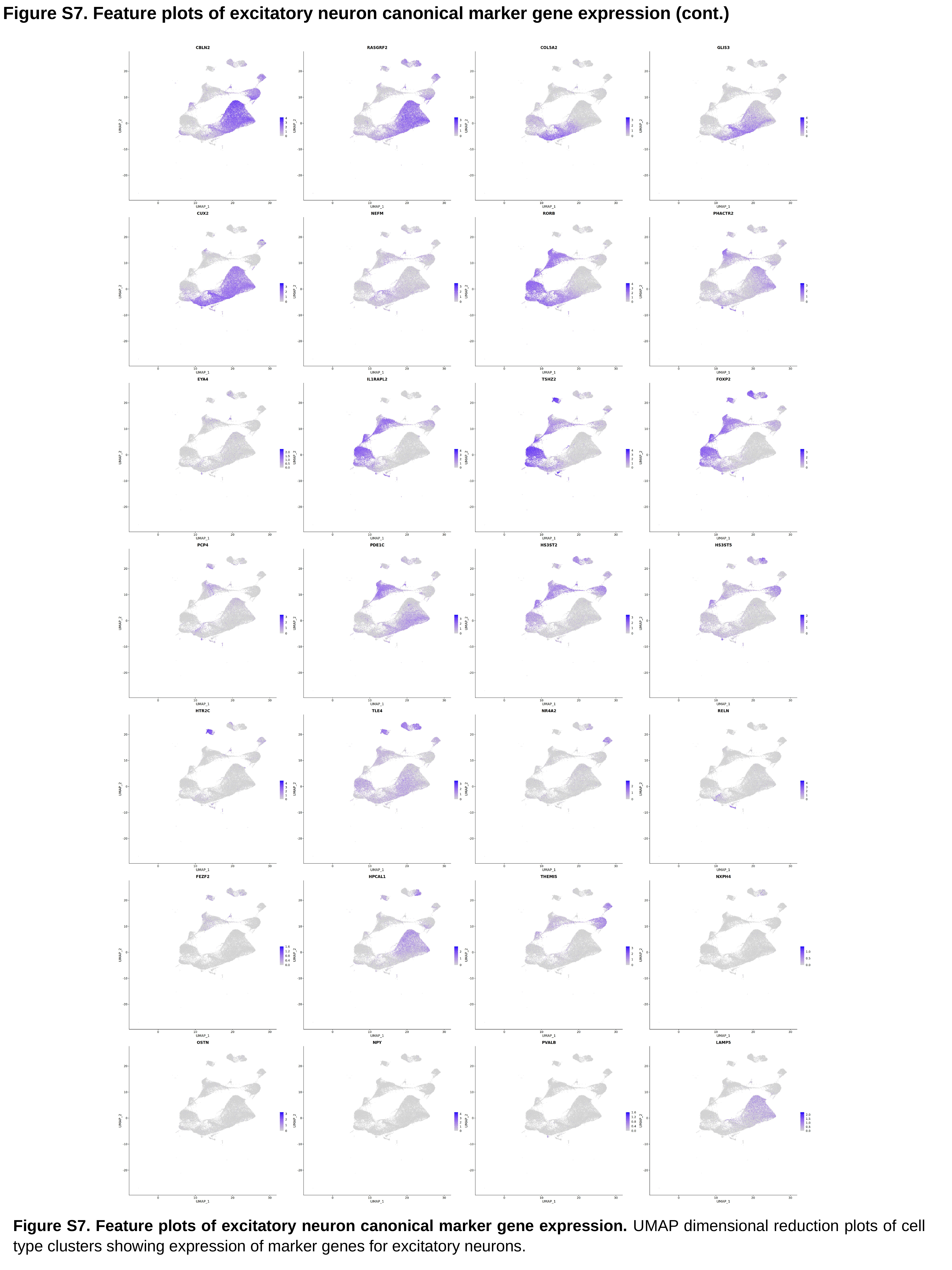

Figure S7. Feature plots of excitatory neuron canonical marker gene expression (cont.)
Figure S7. Feature plots of excitatory neuron canonical marker gene expression. UMAP dimensional reduction plots of cell type clusters showing expression of marker genes for excitatory neurons.

## Slide 12
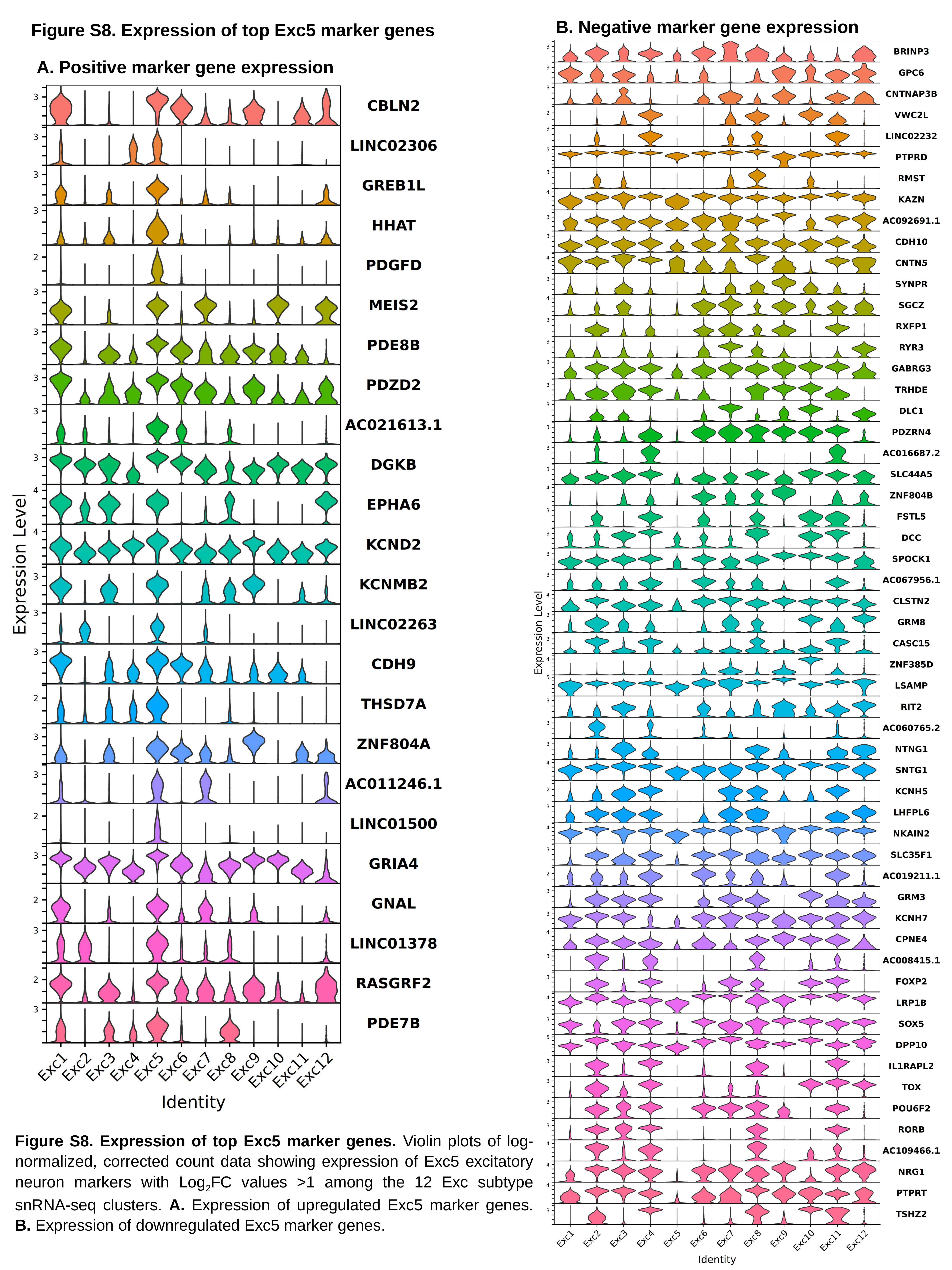

B. Negative marker gene expression
Figure S8. Expression of top Exc5 marker genes
A. Positive marker gene expression
Figure S8. Expression of top Exc5 marker genes. Violin plots of log-normalized, corrected count data showing expression of Exc5 excitatory neuron markers with Log2FC values >1 among the 12 Exc subtype snRNA-seq clusters. A. Expression of upregulated Exc5 marker genes. B. Expression of downregulated Exc5 marker genes.

## Slide 13
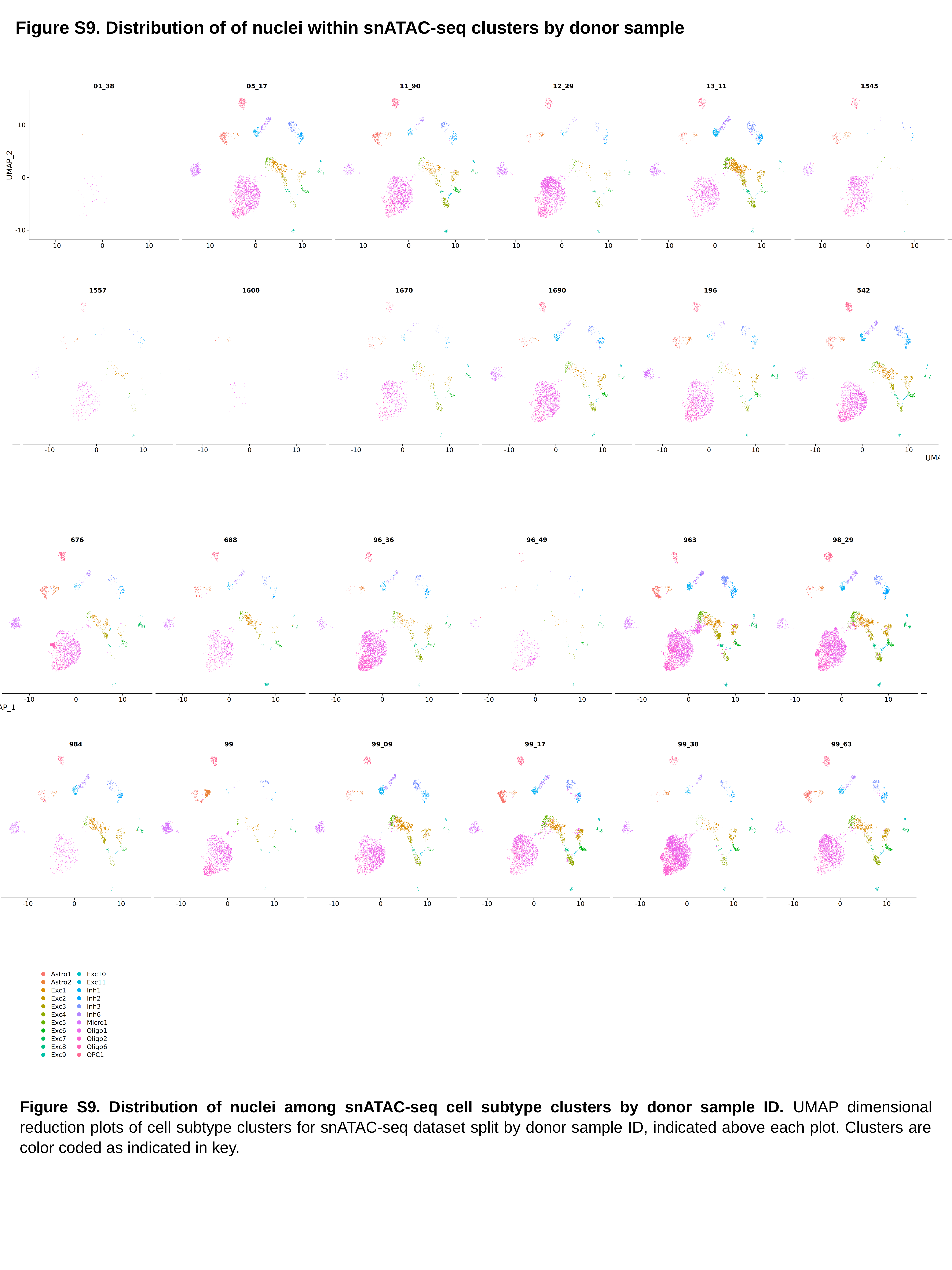

Figure S9. Distribution of of nuclei within snATAC-seq clusters by donor sample
Figure S9. Distribution of nuclei among snATAC-seq cell subtype clusters by donor sample ID. UMAP dimensional reduction plots of cell subtype clusters for snATAC-seq dataset split by donor sample ID, indicated above each plot. Clusters are color coded as indicated in key.

## Slide 14
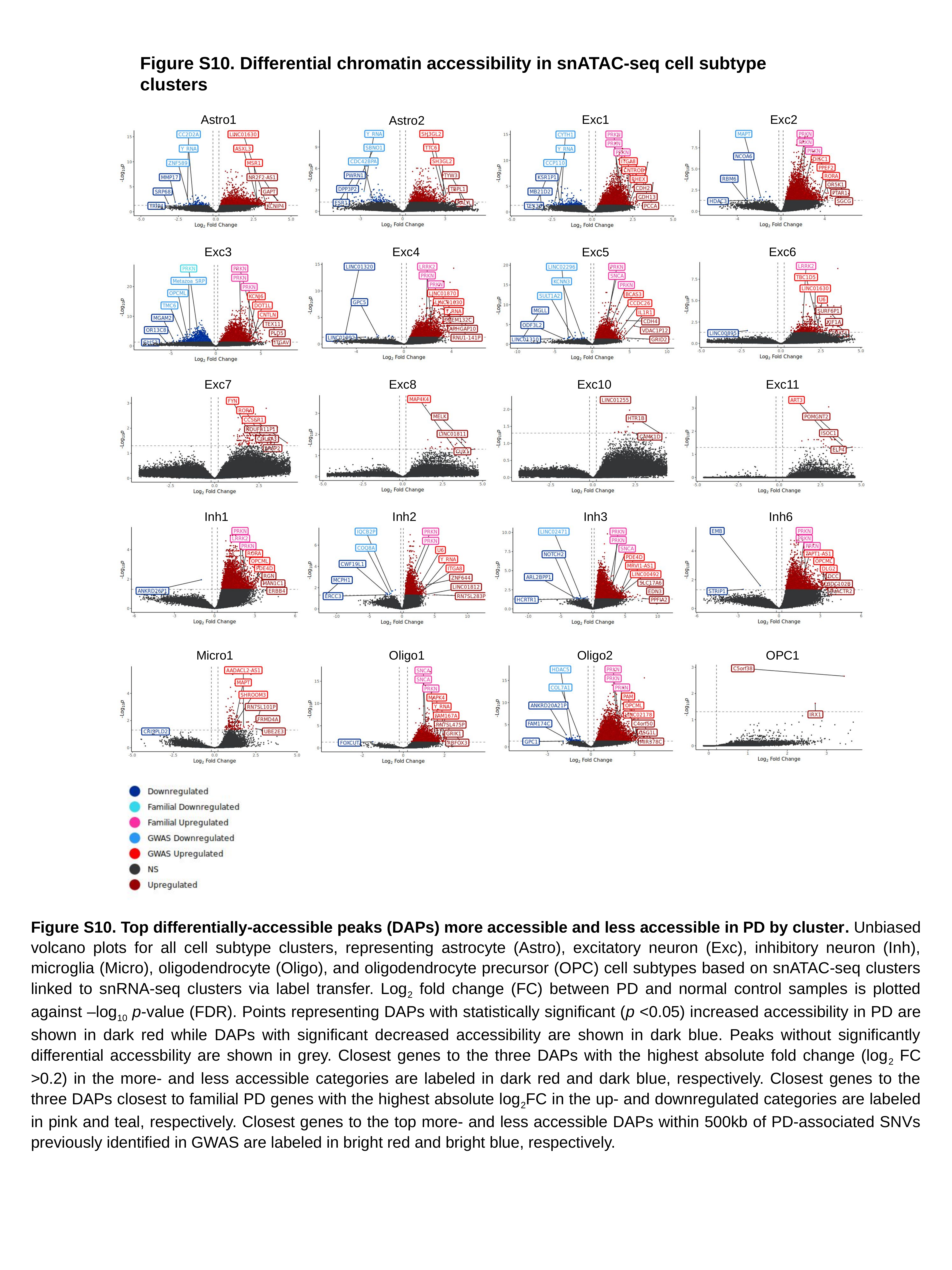

Figure S10. Differential chromatin accessibility in snATAC-seq cell subtype clusters
Astro1
Exc1
Exc2
Astro2
Exc3
Exc4
Exc6
Exc5
Exc7
Exc8
Exc10
Exc11
Inh1
Inh2
Inh3
Inh6
Micro1
Oligo1
Oligo2
OPC1
Figure S10. Top differentially-accessible peaks (DAPs) more accessible and less accessible in PD by cluster. Unbiased volcano plots for all cell subtype clusters, representing astrocyte (Astro), excitatory neuron (Exc), inhibitory neuron (Inh), microglia (Micro), oligodendrocyte (Oligo), and oligodendrocyte precursor (OPC) cell subtypes based on snATAC-seq clusters linked to snRNA-seq clusters via label transfer. Log2 fold change (FC) between PD and normal control samples is plotted against –log10 p-value (FDR). Points representing DAPs with statistically significant (p <0.05) increased accessibility in PD are shown in dark red while DAPs with significant decreased accessibility are shown in dark blue. Peaks without significantly differential accessbility are shown in grey. Closest genes to the three DAPs with the highest absolute fold change (log2 FC >0.2) in the more- and less accessible categories are labeled in dark red and dark blue, respectively. Closest genes to the three DAPs closest to familial PD genes with the highest absolute log2FC in the up- and downregulated categories are labeled in pink and teal, respectively. Closest genes to the top more- and less accessible DAPs within 500kb of PD-associated SNVs previously identified in GWAS are labeled in bright red and bright blue, respectively.
